# Improving socioeconomic status may reduce the burden of malaria in sub Saharan Africa: A systematic review and meta-analysis

**DOI:** 10.1371/journal.pone.0211205

**Published:** 2019-01-24

**Authors:** Abraham Degarege, Kristopher Fennie, Dawit Degarege, Shasank Chennupati, Purnima Madhivanan

**Affiliations:** 1 Department of Epidemiology, Robert Stempel College of Public Health & Social Work, Florida International University, Miami, FL, United States of America; 2 Aklilu Lemma Institute of Pathobiology, Addis Ababa University, Addis Ababa, Ethiopia; 3 Ethiopian Ministry of Health Office, Addis Ababa, Ethiopia; 4 Public Health Research Institute of India, Mysore, India; Instituto Rene Rachou, BRAZIL

## Abstract

**Background:**

A clear understanding of the effects of housing structure, education, occupation, income, and wealth on malaria can help to better design socioeconomic interventions to control the disease. This literature review summarizes the relationship of housing structure, educational level, occupation, income, and wealth with the epidemiology of malaria in sub-Saharan Africa (SSA).

**Methods:**

A systematic review and meta-analysis was conducted following the preferred reporting items for systematic reviews and meta-analyses guidelines. The protocol for this study is registered in PROSPERO (ID=CRD42017056070), an international database of prospectively registered systematic reviews. On January 16, 2016, available literature was searched in PubMed, Embase, CINAHL, and Cochrane Library. All but case studies, which reported prevalence or incidence of *Plasmodium* infection stratified by socioeconomic status among individuals living in SSA, were included without any limits. Odds Ratio (OR) and Relative Risk (RR), together with 95% CI and p-values were used as effect measures. Heterogeneity was assessed using chi-square, Moran’s I^2^, and tau^2^ tests. Fixed (I^2^<30%), random (I^2^≥30%) or log-linear dose-response model was used to estimate the summary OR or RR.

**Results:**

After removing duplicates and screening of titles, abstracts, and full text, 84 articles were found eligible for systematic review, and 75 of them were included in the meta-analyses. Fifty-seven studies were cross-sectional, 12 were prospective cohort, 10 were case-control, and five were randomized control trials. The odds of *Plasmodium* infection increased among individuals who were living in poor quality houses (OR 2.13, 95% CI 1.56–3.23, I^2^ = 27.7), were uneducated (OR 1.36, 95% CI 1.19–1.54, I^2^ = 72.4.0%), and were farmers by occupation (OR 1.48, 95% CI 1.11–1.85, I^2^ = 0.0%) [p<0.01 for all]. The odds of *Plasmodium* infection also increased with a decrease in the income (OR 1.02, 95% CI 1.01–1.03, tau^2^<0.001), and wealth index of individuals (OR 1.25, 95% CI 1.18–1.35, tau^2^ = 0.028) [p<0.001 for both]. Longitudinal studies also showed an increased risk of *Plasmodium* infection among individuals who were living in poor quality houses (RR 1.86, 95% CI 1.47–2.25, I^2^ = 0.0%), were uneducated (OR 1.27, 1.03–1.50, I^2^ = 0.0%), and were farmers (OR 1.36, 1.18–1.58) [p<0.01 for all].

**Conclusions:**

Lack of education, low income, low wealth, living in poorly constructed houses, and having an occupation in farming may increase risk of *Plasmodium* infection among people in SSA. Public policy measures that can reduce inequity in health coverage, as well as improve economic and educational opportunities for the poor, will help in reducing the burden of malaria in SSA.

## Introduction

In 2005, WHO proposed a goal to reduce the incidence of malaria, caused by *Plasmodium* infection, in endemic regions by 75% by 2015 [1]. In order to achieve this goal, between 2000 and 2015, a number of malaria control measures were implemented in regions where transmission of the disease was high. These control measures caused a 37% decrease in incidence, and a 60% decrease in the mortality rate (6.2 million lives saved) related to the disease [1]. However, malaria remains a common public health problem in developing countries. In 2016 alone, approximately 216 million people were infected, and an estimated 445,000 died due to malaria globally [1]. About 90% of these malaria cases and 91% of deaths due to the disease were in sub-Saharan Africa (SSA) [2]. Moreover, in 2013, post-natal mortality from malaria in SSA was estimated to be 437,000 [1]. More than 70% of deaths due to malaria in SSA in 2016 occurred in children under five [2].

The presence of insecticide resistant mosquitoes (*Anopheles gambiae* complex) and favorable climate and environmental conditions, conducive to vector survival, makes malaria transmission high in SSA [3–5]. Moreover, *P*. *falciparum*, which contributes to severe symptoms and high death rates, is the most dominant species in SSA [6, 7]. Furthermore, socioeconomic factors including education, employment, income, and household likely may contribute to the high burden of malaria in SSA [8]. Poorly constructed houses allow easy entrance of *Plasmodium*-carrying vectors and increases chances of infection among family members [9]. Individuals with low income cannot easily afford to buy chemicals to spray in the houses, insecticide treated bed nets (in countries where not provided for free), and drugs or other related medical costs [10–12]. Lack of education also may be related to low levels of awareness about malaria prevention and treatment strategies [13].

Existing literature reports heterogeneous results concerning the effect of house structure, education level, occupation, income, and wealth on malaria. Two previous reviews summarized information about the relationship of malaria with socio economic status (SES) worldwide [14, 15]. However, they summarized the relationship of malaria with wealth [14] and house [15] only and did not consider other SES indicators. In addition, the review by Tusting *et al*. (2013) [14] involved children up to 15 years of age only, and the meta-analysis was limited to comparisons between the least poor (highest) and poorest (lowest) wealth groups. Although the majority of deaths due to malaria occur in children, a considerable proportion of adults also experience malaria infection as well as related morbidities and deaths [16,17]. Adults also can serve as a reservoir/source of infection for malaria in children by carrying low-density parasites for long periods [16]. Moreover, the impact of SES indicators on health may vary across different population age groups [18,19]. Thus, knowing the socioeconomic risk factors of malaria in adults would be useful in designing strategies to control the disease in this population. As various SES indicators may measure different aspects of health risk related to malaria, a systematic examination of the relationship of a variety of SES indicators separately with malaria is important to better understand the indicators that can explain malaria risk and recommend appropriate malaria prevention strategies accordingly. In addition, while Tusting *et al*. (2015) [15] reviewed literature on the effects of housing structure on malaria risk that were published before 2014, several new articles have been published on the topic since. Thus, updating the literature on the subject is warranted to continue to inform policies related to malaria prevention and control measures in endemic regions. The current review summarizes literature on the relationship and effect of house structure (wall, floor, roof, window, eaves, and ceilings), education level (none, primary, secondary or more), occupation, income, and wealth on the epidemiology of malaria in all age groups in SSA.

## Methods

### Protocol and registration

This systematic review and meta-analysis was conducted following the Preferred Reporting Items for Systematic Reviews and Meta-analyses (PRISMA) guidelines (S1 Table) [20]. The protocol for this review is registered in PROSPERO International prospective register of systematic reviews (http://www.crd.york.ac.uk/PROSPERO/display_record.asp?ID=CRD42017056070).

### Inclusion and exclusion criteria

Cross-sectional, case-control, cohort, and randomized control trial (RCT) studies, which reported prevalence or incidence of malaria stratified by SES among individuals living in SSA were included. Conference abstracts, protocols, Gray literature, review papers, commentary reports, unpublished studies, case studies, *in vitro* and animal studies were excluded. Studies that diagnosed malaria based on fever or self-reports without confirming by microscopy, rapid diagnostic test, or polymerase chain reaction were excluded. In addition, studies were excluded from meta-analysis when data on malaria prevalence/incidence were not reported being stratified based on SES status.

### Outcome and exposure measures

Malaria was assessed as the prevalence or incidence of *Plasmodium* infection and SES was assessed in terms house structure (wall, roof, floor, window, ceiling, and eaves), education status (none/illiterate, primary, secondary or above), income level, occupation (farmers, traders, civil servants, students, entrepreneurs etc.), and wealth (poorest, less poor, poor, average, least poor).

### Search methods for identification of studies

On January 16, 2016 available literature was searched in PubMed, Embase, CINAHL and Cochrane Library using keywords (malaria OR *Plasmodium*) AND (“socioeconomic status” OR “socioeconomic position” OR income OR wealth OR poverty OR equity OR house* OR employment* OR occupation* OR education*) (S2 Table). No restriction was imposed on language, age/sex of the study participants, study area, and study design during the literature search. References of the review articles on malaria and SES were hand searched for additional articles [14,15, 21]. Following exclusion of duplicates, the tittles and abstracts were screened simultaneously, and a second round of screening of full texts was conducted on the articles considered eligible by the first screen of title and abstracts based on the inclusion/exclusion criteria. Finally, full text articles to be included in the review were approved. Two authors (AD and SC) searched the literature, screened, and selected the final articles to be included. Any discrepancies in choice of articles included in the review were resolved by consensus between the two authors (AD and SC).

### Data collection

Data on author, year of publication, study country, study design, sample size, crude or adjusted odds ratio (OR) or relative risk (RR) of malaria along with 95% confidence interval (CI) among individuals with different SES were collected. In addition, when appropriate measures of association (OR, RR) were not reported, raw data for prevalence or incidence of malaria among individuals with different SES were extracted from tables, figures, texts, or summary data in the articles. When sufficient data were not available from the articles, raw data for prevalence or incidence of malaria stratified by SES were obtained directly from authors [22–24]. This information was used to estimate the crude ORs or RRs of malaria among individuals with different SES. Data were abstracted and entered into an Excel spreadsheet by two authors (AD and DD) independently, and compared. The two authors discussed and approved the final data used for analysis.

### Quality and bias assessment

Quality and risk of bias of the studies were evaluated on the basis of selection of study participants, study design, assessment of confounders, blinding, data collection methods, and withdrawals and drop-outs using the Effective Public Health Practice Project Tool [25].

### Data analysis

Data were analyzed using Stata software (Version 11, Texas, USA) [26]. ORs and RRs, together with 95% CI were used as effect measures. When studies did not report 95% CI for ORs or RR and when raw data for prevalence or incidence of malaria based on individuals’ SES were not available from tables, figures, texts, or summary data of the articles, we calculated 95% CI from p-values following the method suggested by Altman and Bland [27].

Heterogeneity was assessed using chi-square (with alpha set to .05), Moran’s I^2^ (Inconsistency), and tau2 (τ^2^) for log-linear dose-response model. When Moran’s I^2^ values were ≥ 30%, a random effects model using Der Simonian and Laird method was used to calculate the summary ORs or RRs [28]. When Moran’s I^2^ values were < 30%, a fixed effects model using the inverse variance method was used to estimate the summary OR or summary RR. Publication bias was examined using the Egger test (bias if p<0.1)[29]. Both unadjusted and adjusted OR and RR values were pooled together while estimating the summary OR or RR measures. Alpha of .05, and two-tailed test were used to asses significance of OR and RR. Sources of heterogeneity were evaluated using sub-group analyses. Subgroup analyses were stratified by population sub-groups (children, pregnant, all ages), study design (cross-sectional, case control, prospective cohort, randomized control trials), year of data collection, geographic regions (Eastern, Southern, Western and Central), quality of the study (low, moderate, high). Meta-regression analysis based on random effects models tested the effect of covariates such as population sub-groups, geographic regions, study design, year of data collection, quality of the study, and sample size on the odds or risk of *Plasmodium* infection. Sensitivity analysis assessed the robustness of results by excluding studies with a lower precision.

Some studies assessed the relationship between wealth and malaria by comparing two, three or four better socioeconomic groups to a low baseline group. Thus, the log-linear dose-response model for multiple studies was used to estimate a summary linear trend of OR of *Plasmodium* infection per unit increase in wealth index/socioeconomic group across several studies [30]. Similarly, as the cut-off values used to determine the level of income across studies were not similar, the log-linear dose-response model suggested by Greenland and Longnecker [30] was used to pool the OR of *Plasmodium* infection compared between individuals with different income level across several studies.

## Results

### Search results and characteristics of the included studies

**Fig 1:**
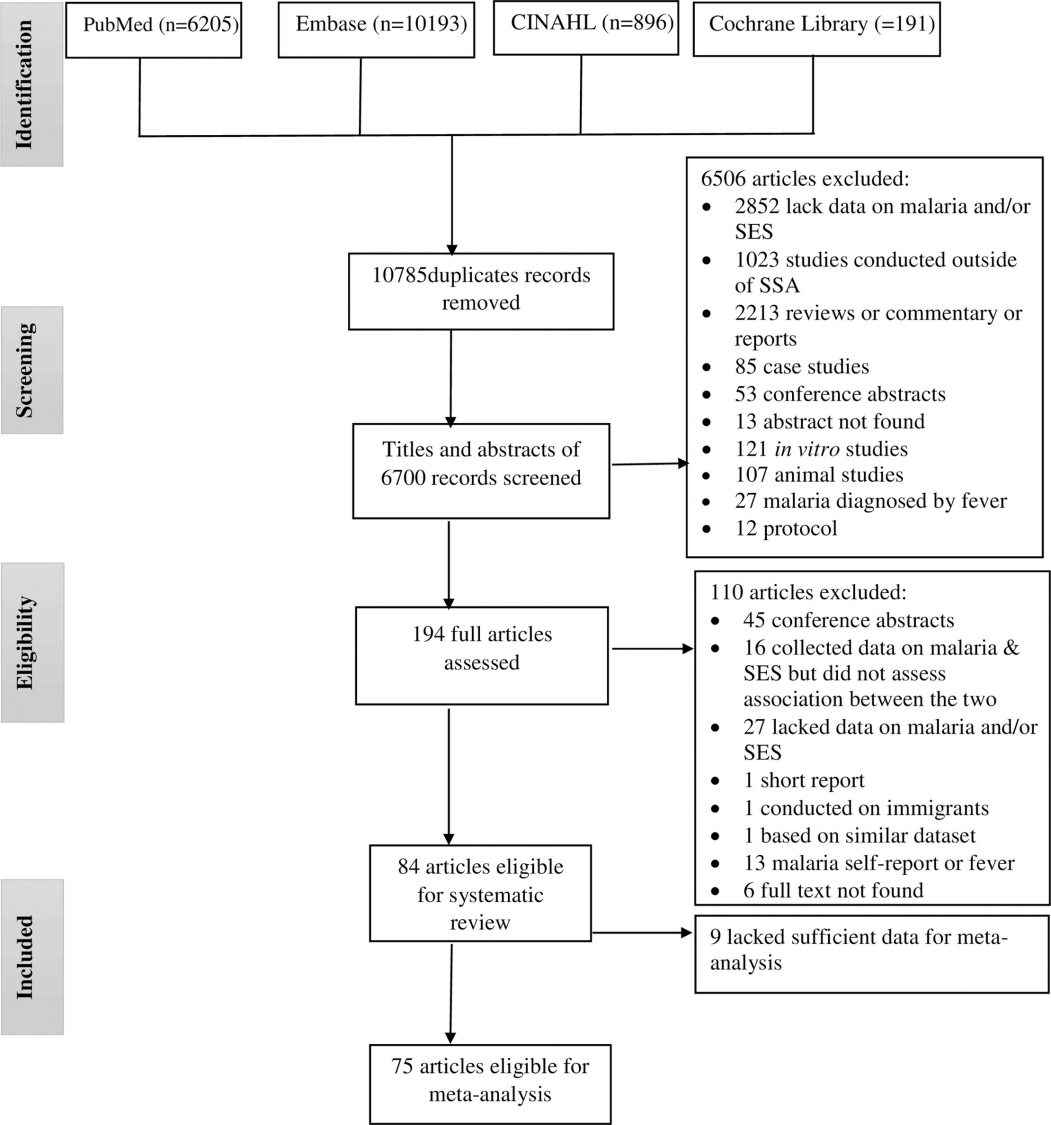
PRISMA flow diagram showing the number of articles retrieved, screened, excluded, and included at each stage of the search of published articles examining the relationship of socioeconomic status with the epidemiology of malaria in sub-Saharan Africa.

A total of 17,485 articles were obtained after searching literature from four databases: Embase (n = 10,193), PubMed (n = 6205), CINAHL (n = 896) and Cochrane Library (n = 191) (Fig 1). Of the 17,485 articles, 10,785 were found to be duplicates. After screening the titles and abstracts of the remaining 6,700 articles, 194 were determined eligible for full text review. A total of 110 articles were excluded after full text review based on the inclusion/exclusion criteria. While 84 articles were included in the systematic review, 75 of them were included in the meta-analysis (S3 Table) [22–24, 31–111]. The studies were conducted in 22 countries in SSA. Out of the 84 studies, 57 were cross-sectional, 12 were prospective cohort, 10 were case-control and five were randomized control trials. Participants were exclusively children in the majority of studies.

### Housing structure and malaria

**Fig 2:**
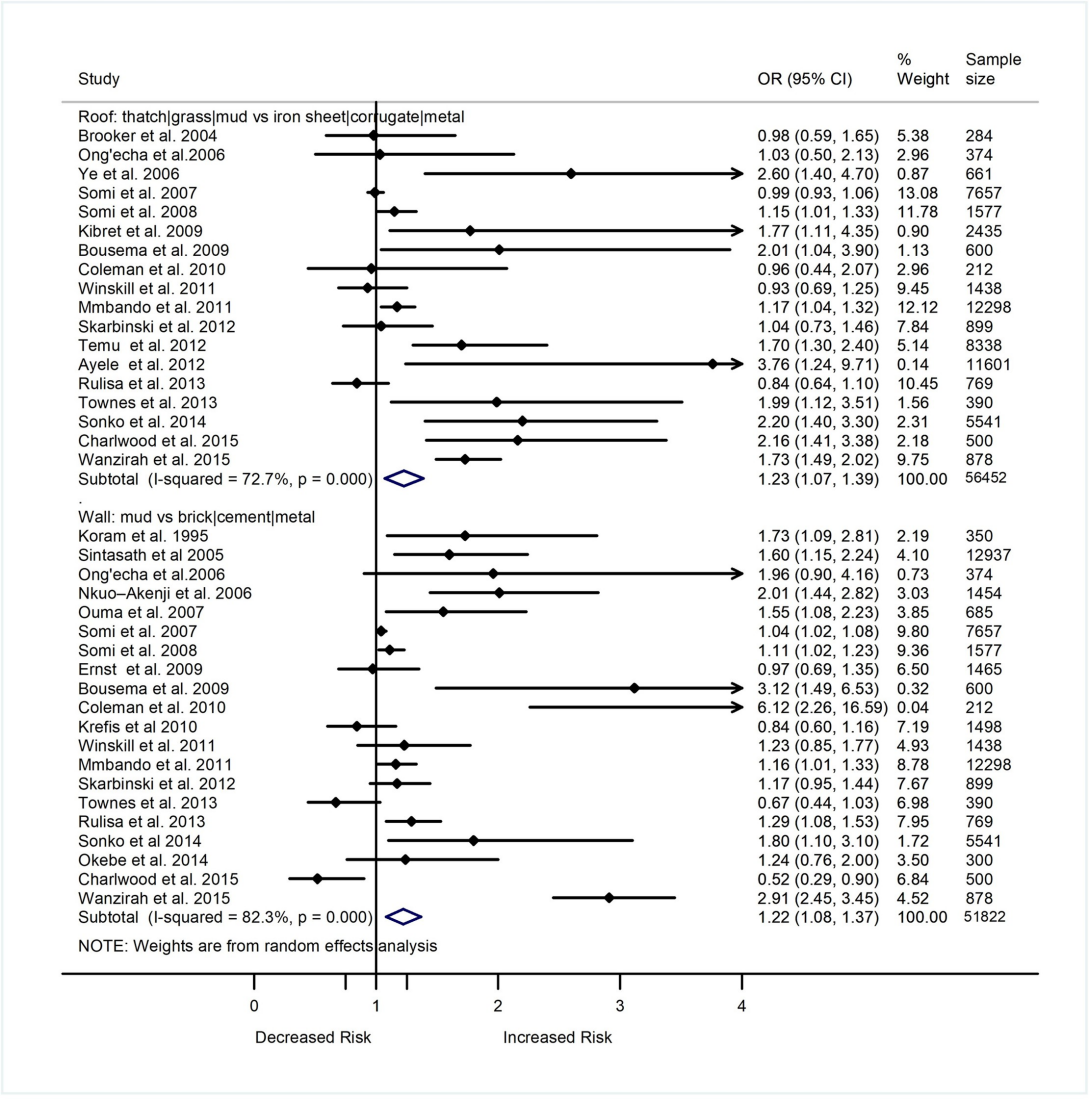
Forest plot showing the relationship of the roof and walls nature of a house with the epidemiology of malaria in sub-Saharan Africa. Values show the odds ratio of *Plasmodium* infection (95% CI). Subtotal (summary) ORs estimated using random effect models. Weights estimated using inverse variance method. I^2^, a measure of heterogeneity.

**Fig 3:**
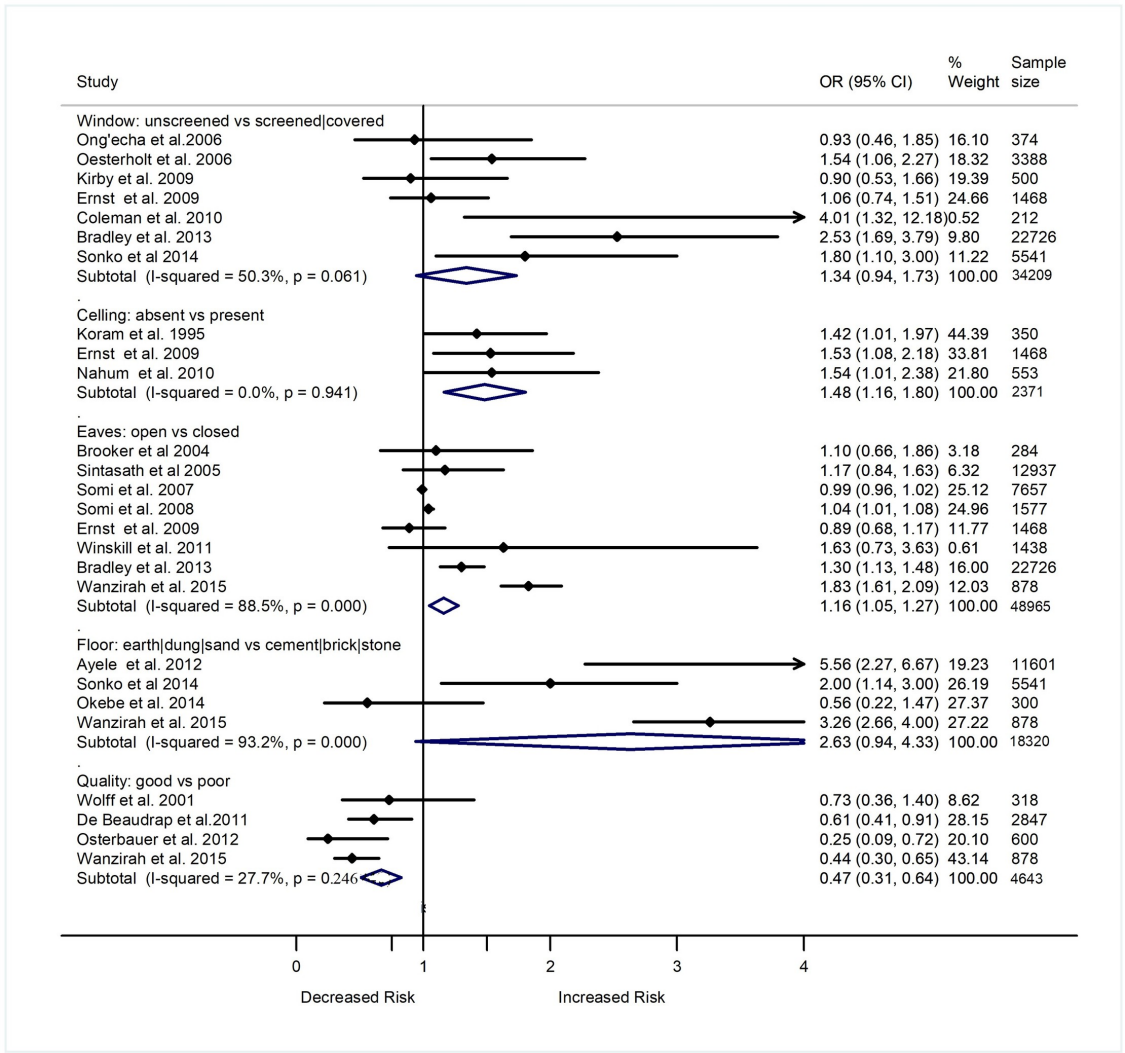
Forest plot showing the relationship of windows, floor, ceiling and eaves nature of a house with the epidemiology of malaria in sub-Saharan Africa. Values show the odds ratio of *Plasmodium* infection (95% CI). Subtotal (summary) ORs estimated using random effect models when I^2^ ≥30 and using fixed effect models when I^2^ <30. Weights estimated using inverse variance method. I^2^, a measure of heterogeneity.

Out of 84 studies included in this review, 40 assessed association of the house structure wall (n = 22), roof (n = 19), window (n = 11), floor (n = 7), eaves (n = 10), ceiling (n = 3)) with prevalence or incidence of malaria in SSA. In terms of design, 19 were cross-sectional, 10 were cohort, eight were case-control, and two were RCTs. All 40 studies were included in the meta-analyses that summarize study findings on the relationship of house structure with malaria. Figs 2 and 3 show study findings comparing the odds of *Plasmodium* infection among individuals living in houses with mud walls (n = 20/22), thatch/grass/mud roofs (n = 18/19), unscreened/uncovered windows (n = 7/11), earth/dung floors (n = 4/7), lack of ceilings (n = 3/3), open eaves (n = 8/10), of poor quality in general (n = 4/9) versus those who were living in houses with cement/corrugated/metal walls, iron sheet/tile roofs, screened/covered windows, cement/brick/stone floors, ceilings present, closed eaves, and of good quality, respectively. Majority of the studies included in the meta-analyses reported increased odds of malaria among individuals living in houses with mud walls (n = 12/20), thatch/grass/mud roofs (n = 11/18), earth/mud/sand floors (n = 3/4), unscreened/uncovered windows (n = 4/7), open eaves (n = 3/8), and lack of ceilings (n = 3/3). However, some studies reported lack of association between the prevalence or incidence of malaria and the type of walls (n = 7/20), roofs (n = 7/18), windows (n = 3/7), eaves (n = 5/8), and floors (n = 1/4) of houses in which individuals were living. One study reported decreased odds of malaria among individuals living in houses with mud walls. A summary analysis of the studies showed increased odds of *Plasmodium* infection among individuals living in houses with thatch/grass/mud roofs (OR 1.23, 95% CI 1.07–1.39, p = 0.002, I^2^ = 72.7%), mud walls (OR 1.22, 95% CI 1.08–1.37, p = 0.001, I^2^ = 82.3%), earth/mud/sand floors (OR 2.63, 95% CI 0.94–4.33, p = 0.054, I^2^ = 93.2%), unscreened window/uncovered windows (OR 1.34, 95% CI 0.94–1.73, p = 0.052, I^2^ = 50.3%), open eaves (OR 1.16, 95% CI 1.05–1.27, p<0.001, I^2^ = 88.5%), and lack of ceilings (OR 1.48, 95% CI 1.16–1.80, p<0.001, I^2^ = 0.0%) compared to those who were living in houses with iron sheet/tile roofs, cement/corrugated walls, cement floors, screened windows, closed eaves, and ceilings, respectively (Figs 2 and 3).

Rather than looking at the independent effect of windows, roofs, walls, eaves, and ceilings on malaria, some studies assessed the combined effect of these structures. Using these structures, studies grouped houses as poor quality (thatched roofs, dirt floors, completely uncovered windows, no ceilings, rough or mud walls and open eaves), and high quality (iron/tile roofs, concrete/brick walls, closed eaves, screened windows and ceilings). A summary analysis of four cross-sectional studies showed increased odds of malaria among individuals living in poor quality houses compared to those living in high quality houses (OR 2.13, 95% CI 1.67–2.94, p<0.001, I^2^ = 5.4%) (Fig 3). A summary analysis of five cohort studies also showed increased risk of malaria among individuals living in poor quality houses compared to those living in high quality houses (RR 1.86, 95% CI 1.47–2.25, p<0.001, I^2^ = 0.0%) (S1 Fig). A meta-analysis of two studies also showed greater incidence of malaria among individuals living in houses with open eaves compared to those living in houses with closed eaves (RR 1.62, 95% CI 1.26–1.98, p<0.001, I^2^ = 0.0%) (S1 Fig).

### Education and malaria

**Fig 4:**
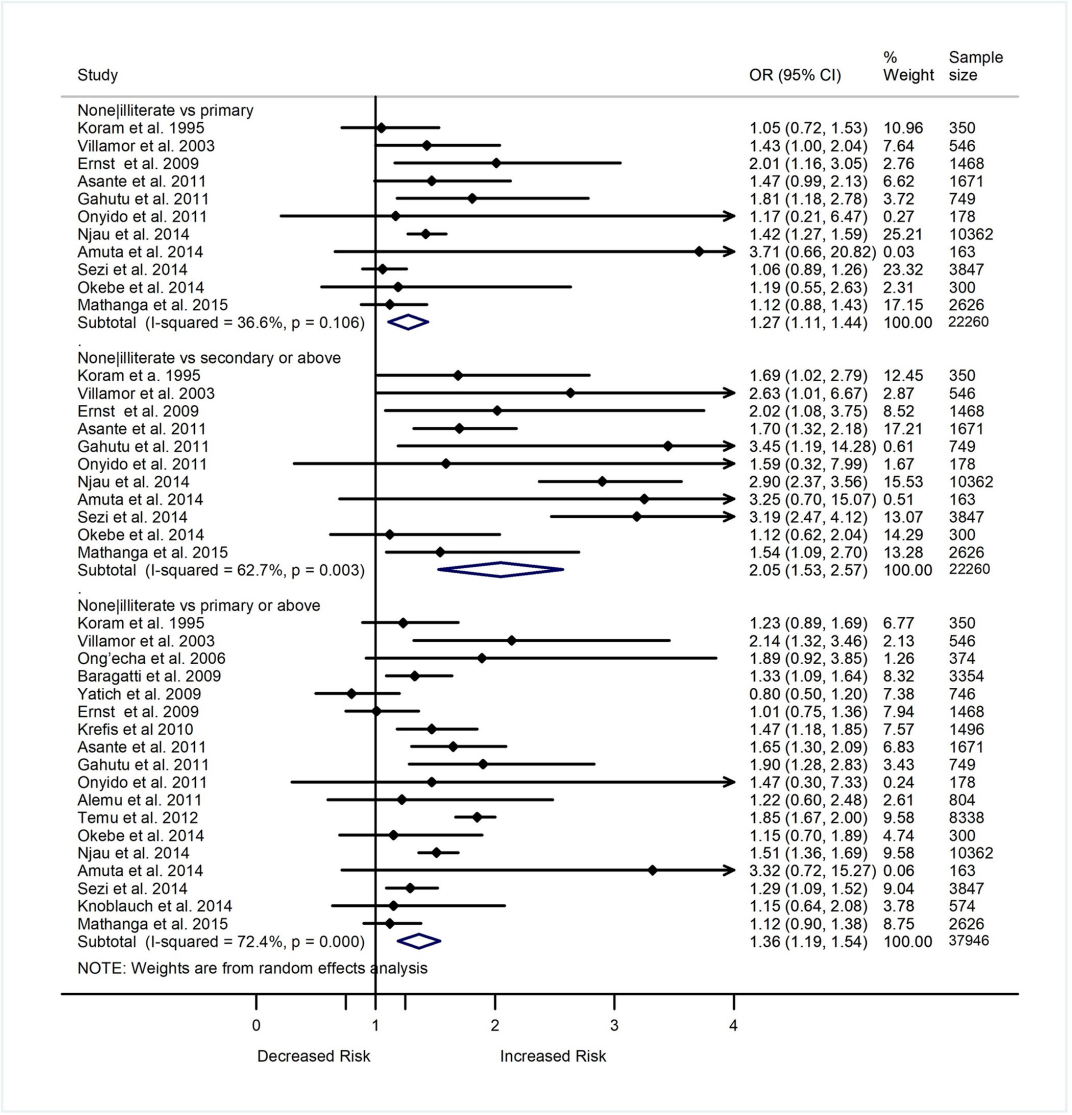
Forest plot comparing the odds ratio of *Plasmodium* infection between individuals without formal education or illiterate with those who had primary and secondary or more education level. Subtotal (summary) ORs estimated using random effect model. Weights estimated using inverse variance method. I^2^, a measure of heterogeneity.

**Fig 5:**
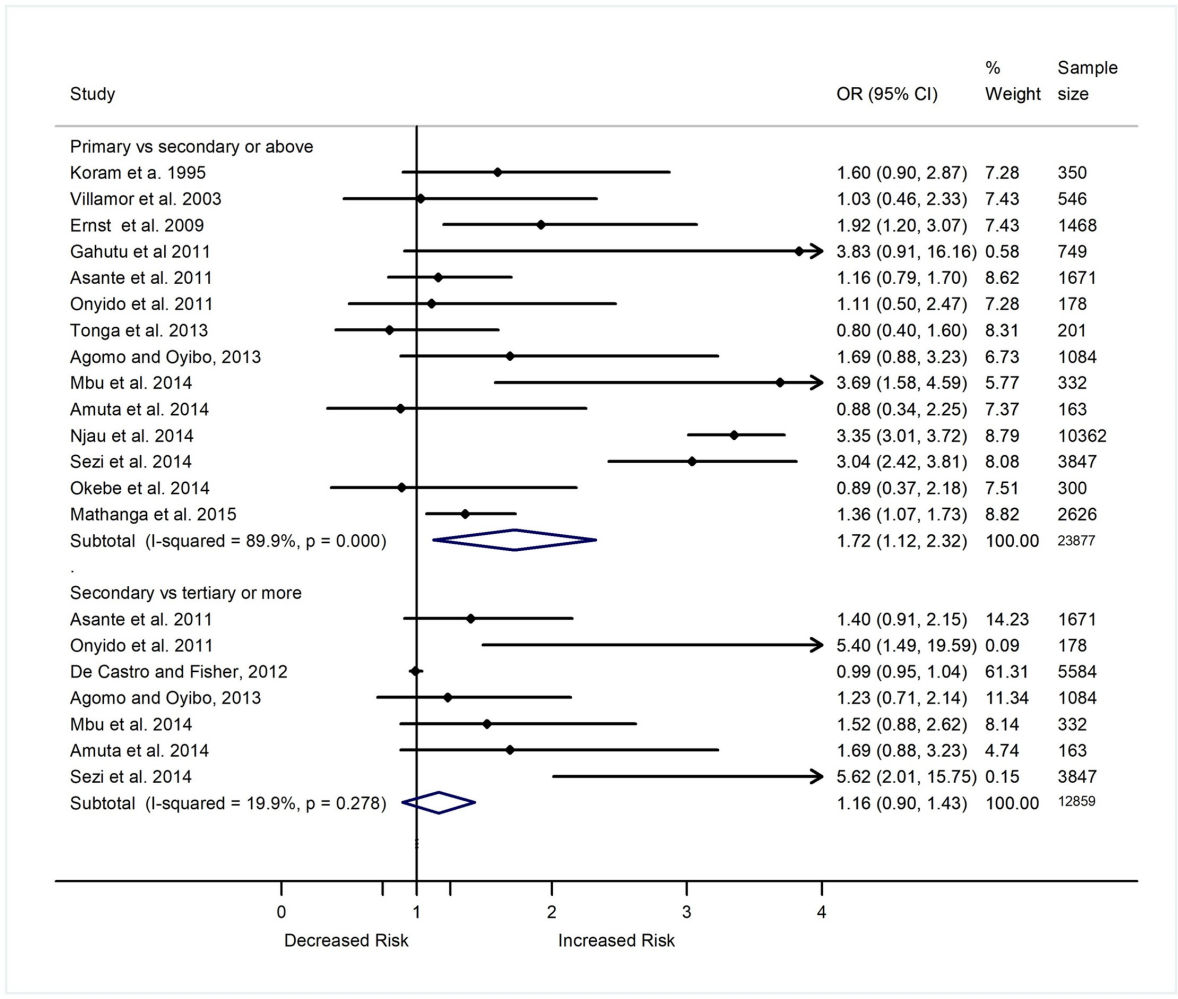
Forest plot comparing the odds ratio of *Plasmodium* infection between individuals with primary education level versus those with secondary or more, and those with secondary versus tertiary or more education level Subtotal (summary) ORs estimated using random effect models when I^2^ ≥30 and using fixed effect models when I^2^<30. Weights estimated using inverse variance method. I^2^, a measure of heterogeneity.

A total of 29 studies included in this review assessed the nature of relationship of education status with the prevalence or incidence of malaria. Of the 29 studies, 23 were included in the meta-analysis that estimated the relationship between education and the odds of *Plasmodium* infection. Fig 4 shows reports of 18 studies comparing the odds of *Plasmodium* infection between individuals who had no formal education or were illiterate versus those who had formal education (primary or more). Out of the 18 studies, eight showed greater odds of *Plasmodium* infection among individuals with no formal education or were illiterate than those with primary or more education level. A meta-analysis of the 18 studies showed higher odds of *Plasmodium* infection among uneducated/illiterate individuals (adults or children with uneducated/illiterate parents) as compared to educated individuals (OR 1.36, 95% CI 1.19–1.54, p<0.001, I^2^ = 72.4%) (Fig 4). The increased odds of *Plasmodium* infection among uneducated/illiterate individuals compared to those educated remained significant after stratifying the data (11 studies) based on level of education: primary level (OR 1.27, 95% CI 1.11–1.44, p<0.001, I^2^ = 36.6%), and greater than primary level (OR 2.05, 95% CI 1.53–2.57, p<0.001, I^2^ = 62.7%) (Fig 4). When compared to individuals with greater than primary education level, those with primary education level showed greater odds of *Plasmodium* infection (OR 1.72, 95% CI 1.12–2.32, p = 0.004, I^2^ = 89.9%, 14 studies) (Fig 5). However, the summary odds of *Plasmodium* infection were comparable between individuals with secondary education versus those with higher than secondary education level (OR 1.16, 95% CI 0.90–1.43, p = 0.208, I^2^ = 19.9%, 7 studies) (Fig 5).

Different longitudinal cohort studies and RCTs also showed that education status could affect the risk of malaria. A summary analysis based on two longitudinal cohort studies showed an increased risk of malaria in children who had illiterate/uneducated mothers compared to those who had mothers with primary or greater educational level (RR 1.27, 95% CI 1.03–1.50, p = 0.013, I^2^ = 0.0%) [68,70]. Another longitudinal study in Uganda also showed lower risk of malaria among children whose parents had more than primary education compared to those with families who were illiterate or had a primary level of education (Incidence Rate Ratio [IRR] 0.78, 95% CI 0.62–0.99, p = 0.038) [94]. Similarly, an RCT in Ghana found a significant decrease in the prevalence of malaria (30.9% to 10.3%, p = 0.003) among children who received education about the transmission and prevention mechanisms of the disease, but the prevalence of the disease increased from 9.5% to 15.9% in those who did not receive the education [38]. Another RCT in Ghana showed an association between some education and a reduction in the risk of high density parasitaemia among pregnant women (OR 0.72, 95% CI 0.54–0.96, p = 0.025) [47]. Ethiopian children whose parents received education about use of insecticide treated bed nets (ITN) were found to be at a lower risk of *Plasmodium* infection compared to those who did not have the training (OR 0.42, 95%CI 0.32–0.57, p<0.001) [51].

However, a study conducted in Ethiopia showed lack of association between education status and the odds of *Plasmodium* infection [64]. As the study lacked sufficient data, it was excluded from the meta-analysis evaluating the relationship between education and prevalence or incidence of malaria.

### Occupation and malaria

**Fig 6:**
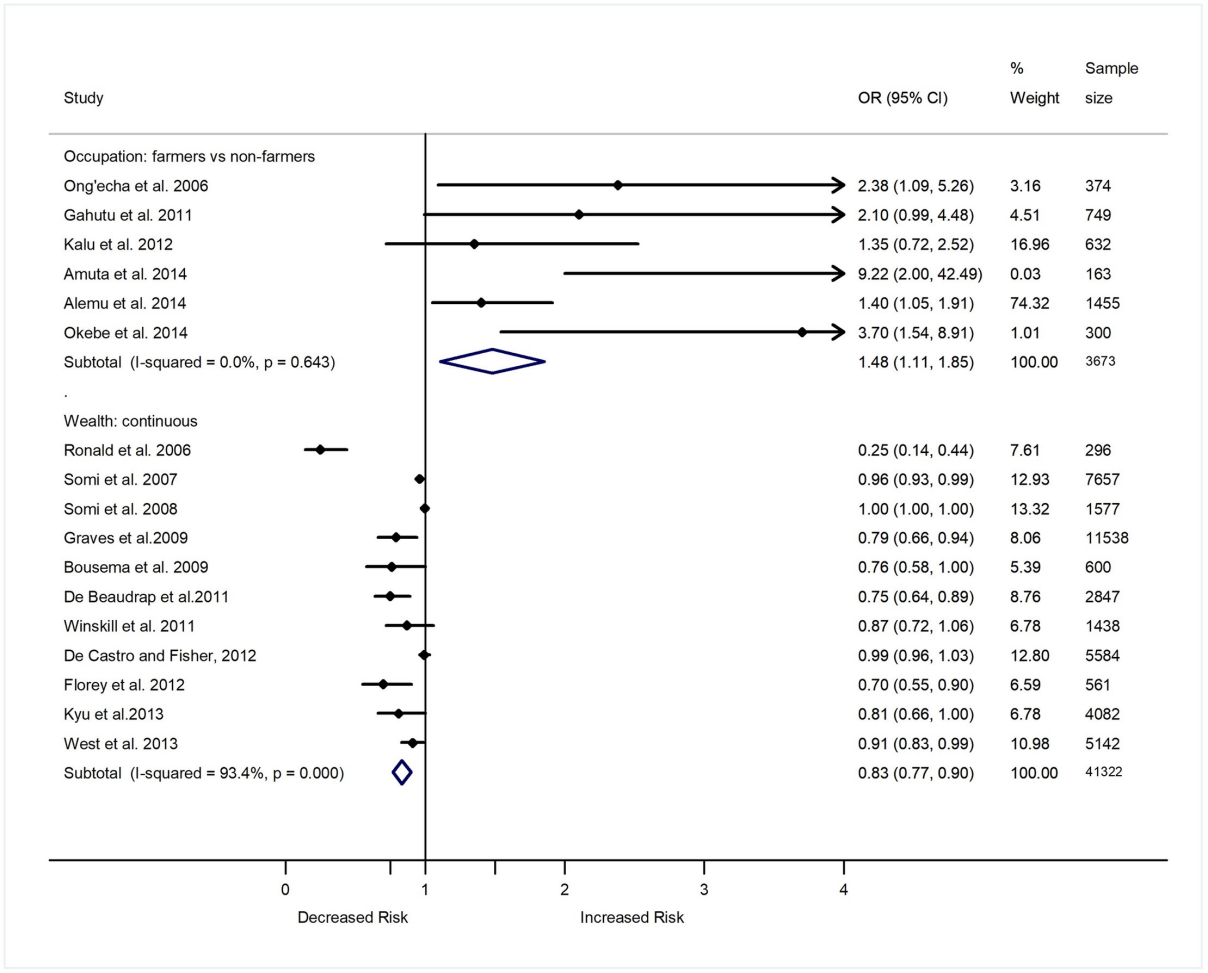
Forest plot showing the relationship of wealth and occupation with the epidemiology of malaria in sub-Saharan Africa. Wealth was treated as continuous and occupation was treated as a categorical variable. Values show the odds ratio of *Plasmodium* infection (95% CI). Total (summary) ORs estimated using random effect model. Weights estimated using inverse variance method. I-Squared, a measure of heterogeneity.

Malaria burden also is posited to be linked with the occupation of individuals. Eleven studies assessed if the type of occupation of individuals is associated with the risk of contracting malaria in seven SSA countries. Seven studies were cross-sectional [33,34,50,64,55,62,83], two were case-control [82,79], and two were longitudinal in design [61,68]. Six of the studies compared the odds or prevalence of malaria between individuals with agricultural related activities (farmers) and others (civil servants, entrepreneurs, traders, students). A summary analysis based on the six studies (three cross-sectional and three case-control) showed that the odds of *Plasmodium* infection increased among farmers, or children with farming parents than individuals with non-agriculture related occupations (OR 1.48, 95% CI 1.11–1.85, p = 0.003, I^2^ = 0.0%) (Fig 6). The study by Alemu *et al*. (2014) [33] contributed close to ¾ of the weight of the summary estimates of the studies that compared the odds of *Plasmodium* infection among farmers, or children with farming parents’ versus those with a non-agriculture related occupation. Another longitudinal study among infants in Ghana also showed an increased incidence of malaria among infants with mothers who farmed compared to infants with mothers who were not farmers (IRR 1.36, 95% CI 1.18–1.58, p<0.001) [68]. A study in Kenya showed an increased risk of malaria among people with outdoor occupation (coefficient = 0.57; 95% CI 0.38–0.76, p<0.0001) [61]. However, De Castro & Fisher (2012) reported lack of significant difference in the prevalence of malaria between farmers and non-farmers in Tanzania (p = 0.450) [50]. As the study by De Castro & Fisher (2012) [50] did not report ORs/RRs estimates or provide raw data on the prevalence/incidence of malaria which was stratified by occupation status, it was excluded from the meta-analysis that estimated the odds of *Plasmodium* infection between farmers and non-farmers. Two studies in Ethiopia and Nigeria also reported a lack of association between the prevalence of malaria and occupation status [64,83].

### Income and malaria

Six studies included in this review reported the nature of the association of income status with the occurrence of *Plasmodium* infection in SSA [32,55,64, 80,100,109]. A high prevalence of *P*. *falciparum* parasitaemia was seen in preschool children coming from households with monthly income <5000 Rwanda Francs (equivalent to <5.92 US dollars) compared to those who came from households with monthly income ≥5000 Rwanda Francs in Rwanda (OR 1.59, 95% CI 1.05–2.40, p = 0.028) [55]. Similarly, a study in Cameroon documented increased odds of *Plasmodium* infection in pregnant women with monthly income <28, 000 CFA franc (equivalent to <51.43 US dollars) compared to those with monthly income >28000 CFA franc (OR 3.9, 95% CI 1.3–11.5, p = 0.014) [100]. The prevalence of malaria in Ghana was also higher among pregnant women with a weekly income< 200,000 Cedis (equivalent to <20 US dollars) compared to those with a weekly income >200,000 Cedis (OR 1.8, 95% CI 1.2–2.9, p = 0.009) [109]. In Sudan, the odds of *Plasmodium* infection among pregnant women with low income was also found to be two times as likely as the odds of infection in pregnant women with high income (OR 2.3, 95% CI 1.3–4.0, p = 0.004) [80]. A study in Ethiopia also found increased odds of *Plasmodium* infection among adults with monthly income <31.25 US dollars compared to those with monthly income >62.5 US dollars (OR 3.67, 95% CI 1.05–12.79, p = 0.041) [32]. However, another study in Ethiopia showcased an increased prevalence of malaria with an increase in annual household income status (regression coefficient = 0.0002, p<0.01) [64].

The cut-off value used was not similar across the five countries to determine the level of income between the comparison groups. Thus, pooling of the five studies was done using the log-linear dose-response model for multiple studies suggested by the Greenland and Longnecker (1992) [30]. A summary estimate based on the five studies showed that a one US dollar decrement in the monthly income of individuals is associated with a 2% increase in the odds of *Plasmodium* infection (OR 1.02, 95% CI 1.01–1.03, p<0.001, τ2 <0.001).

### Wealth and malaria

Rather than looking at the independent effect of housing, education, occupation, and income measures on malaria, some studies assessed the combined effect of these factors. The factors included household construction materials (walls, roofs, floors, windows), possession of assets (car, motorbike, bicycle, fridge, television, radio and mobile phone, electricity at home, bed net, animals), source of water, type of toilet, education, and occupation of household. Studies converted these different indicators of SES into a single socioeconomic score/wealth index using principal component analysis and treated as a single variable (wealth). Thus, the index provided a maximum discrimination in the socioeconomic status of individuals among households. After determining the weights for each socioeconomic indicator in the index using principal components analysis, the scores in the index were grouped into different socioeconomic categories based on their weights. However, the number and type of socioeconomic indicators used to develop the composite index and number of categories of the socioeconomic factor varied among studies. Many studies grouped the wealth index into five (n = 19)(1 = poorest to 5 = least poor) [24,35,36,42,44,52,57,58,63,71,72, 88, 91, 93,95,97,98,104,111] some into four (n = 4) (1 = poorest to 4 = least poor) [48,53,99,108] and some into three (n = 10) (1 = poor, 2 = average, 3 = least poor) [23, 46,60,61,68,76,89,94,103,107] categories (i.e. Socioeconomic groups). Many studies also treated the wealth index as a continuous variable (n = 11) [40,49,50,54,58,69,89,95,96,104,105]. Three of the studies treated wealth both as a categorical and continuous variable [58,95,104]. In sum, 41 studies evaluated the relationship between wealth index and the odds of *Plasmodium* infection. Of the 41 studies, 38 were included in the meta-analyses that summarized the relationship between wealth and malaria.

A summary analysis of 30 studies, which treated wealth index as a categorical variable, showed a 20% reduction in the odds of *Plasmodium* infection with one unit increase in socioeconomic group or wealth index category of individuals (OR 0.80, 95% CI 0.74–0.85, p<0.001, *τ*^2^ = 0.028, Model chi2 = 44.52) (S3 Table) [23,24,35,42,44, 46,48,52,53,57,58,60,63,71,72,76, 88,89, 91, 93–95,97–99,103,104,107,108,111]. A summary analysis of the eleven studies, which treated wealth index as a continuous variable, also showcased that the odds of *Plasmodium* infection decreased by 0.83 unit with every one unit increase in the wealth index (OR 0.83, 95% CI 0.77–0.90, p<0.001, I^2^ = 93.4%) (Fig 6) [40,49,50,54,58,69,89,95,96,104,105]. The remaining two studies, which treated wealth as categorical variable but were not included in the meta-analysis due to lack of sufficient data, also showed a trend of lower odds of *Plasmodium* infection with an increase in the wealth index of individuals [36,57]. Gosoniu *et al*. (2012) [57] reported significantly lower odds of malaria among individuals who were in the least poor (OR: 0.28, 95% CI: 0.17–0.48, p<0.001) or average (OR 0.55, 95% CI 0.42–0.72, p<0.001) socioeconomic category as compared to those in the poorest category. Asante *et al*. (2013) [36] also reported a decreased hazard ratio of *Plasmodium* infection among individuals who were in the least poor socioeconomic group as compared to those who were in the poorest socioeconomic group (OR 0.45, 95% CI 0.36–0.56, p<0.001). However, Homan *et al*. (2016) [61] reported increased prevalence of malaria among individuals with high socioeconomic group as compared to those in the low socioeconomic group (OR 1.27, 95% CI 1.08–1.51, p = 0.005).

### Sensitivity analysis

Most of the summary ORs or RRs showing relationship between SES indicators and malaria prevalence or incidence were not influenced by individual studies. The summary ORs and the corresponding 95% CI values of *Plasmodium* infection comparing individuals based on the nature of the house structure-particularly wall, roofs, window and eaves, education status, occupation and income level did not change after removing one study serially. However, the summary OR and the corresponding 95% CI values estimated in a meta-analysis after including four studies that reported the odds of *Plasmodium* infection among individuals who were living in houses with earth floor versus those living in houses with cement/stone floors (OR 2.63, 95% CI 0.94–4.33) changed to 1.94 (0.22–3.66) after removing the data by Ayele *et al*. (2012) [37] (OR 5.56, 95% CI 2.27–6.67), and to 2.94 (0.53–5.34) after removing the data by Sonko *et al*. (2014) [97] (OR 2.0, 95% CI 1.14–3.00). Similarly, the summary OR and 95% CI of *Plasmodium* infection among individuals who were primary versus those with secondary or above education level estimated after including 14 studies (OR 1.72, 95% CI 1.12–2.32) changed to 1.60 (0.99–2.22) after removing the study by Mbu *et al*. (2014) [73] (OR 3.69, 95% CI 1.58–4.59).

### Heterogeneity and meta-regression analysis

There was no heterogeneity (I^2^ = 0.0%) among the studies that reported the OR of *Plasmodium* infection among individuals living in good versus poor quality houses, houses with ceilings versus lack ceilings, high versus low income, and those with farming versus non-farming occupation. Heterogeneity was low to moderate among the studies that compared the OR of *Plasmodium* infection among individuals living in house with unscreened versus screened/covered windows (I^2^ = 50.3%), uneducated versus primary level (I^2^ = 36.6%), and uneducated versus secondary level (I^2^ = 62.7%). However, there was a high level of heterogeneity among the studies comparing the OR of *Plasmodium* infection based on roof (I^2^ = 72.7%), wall (I^2^ = 82.3%), floor (I^2^ = 93.2%), and eaves (I^2^ = 88.5%) nature of the house, and wealth index (*τ*^2^ = 0.028) of individuals.

Meta-regression analyses showed that geographic region where the studies were conducted significantly associated with the OR of *Plasmodium* infection reported in each individual study that examined relationship of the roof of the house (Meta regression coefficient (β) = -0.39, p = 0.045), education level (β = 0.21, p = 0.002) and wealth status (β = 0.49, p = 0.039) with the prevalence of malaria (S4 Table). Year of data collection was also significantly associated with the OR of *Plasmodium* infection reported in each individual study that examined the relationship of the prevalence of malaria with the wealth index of individuals (β = 0.10, p = 0.041). In addition, study design (β = 0.79, p = 0.042), geographic region (β = 0.63, p = 0.040), age of the study population group (β = -0.15, p = 0.034), and sample size (β = 0.00001, p = 0.016) were significantly associated with the OR of *Plasmodium* infection reported in each individual study that examined the relationship of the prevalence of malaria with the educational status of the study participants. However, only geographic region was statistically significantly associated with the OR of *Plasmodium* infection reported in each individual study that examined the relationship of SES with the prevalence of malaria after adjustment for multiple comparisons using the Bonferroni correction (Bonferroni corrected p = 0.012) (S4 Table).

Indeed, after doing a sub-group analyses, the heterogeneity level decreased among studies that were conducted in West Africa region to examine the relationship of educational status (42.4%), and roof of a house (I^2^ = 66.9%) and wall (I^2^ = 72.4%) with the prevalence or incidence of malaria. The heterogeneity level among the studies that examined the relationship between educational status and malaria prevalence or incidence also decreased after stratifying data based on level of education (primary I^2^ = 36.6%, greater than primary I^2^ = 62.7%, and tertiary or more I^2^ = 24.1%) and study design (cross sectional[I^2^ = 0.0%], case control [I^2^ = 74.7%]). The heterogeneity level among the studies that examined the relationship between the roof a house and malaria prevalence or incidence also decreased after sub-group analyses based on study design (case control [I^2^ = 0.0%], longitudinal [I^2^ = 0.0%], cross sectional [I^2^ = 63.8%]) and quality of the studies (moderate quality (I^2^ = 56.3). Similarly, sub-group analysis based on study design (cross sectional [I^2^ = 67.9%], case control [I^2^ = 30.5%], longitudinal [I^2^ = 97.0%]) and quality of the study (moderate quality (I^2^ = 54.2%) decreased the heterogeneity level among the studies that examined the relationship between the wall of a house and prevalence or incidence of malaria. There was no heterogeneity among studies with case control design that examined the relationship of the nature of eaves of a house with the epidemiology of malaria.

A meta-analysis after grouping the studies based on the age of the study population groups also showed varied results on the relationship between SES indicators and malaria prevalence or incidence in children and adults. The increased odds of *Plasmodium* infection among individuals who were living in houses with mud wall (OR 1.22, 95% CI 1.08–1.37), were uneducated (OR 1.36, 95% CI 1.19–1.54), and were farmers by occupation (OR 1.48, 95% CI 1.11–1.85) was greater in children (mud wall: OR 1.32, 95% CI 1.03–1.62; uneducated: OR 1.45, 95% CI 1.28–1.62; farmers: OR 2.39, 95% CI 1.13–3.65) than in adults (mud wall: OR 1.13, 95% CI 0.96–1.30; uneducated: OR 1.03, 95% CI 0.74–1.32; farmers: OR 1.39, 95% CI 1.00–1.78). Similarly, the decrease in the odds of *Plasmodium* infection with an increase in the odds of wealth status (OR 0.83, 95% CI 0.77–0.90) was greater among children who belong to families with a high wealth status (OR 0.75, 95% CI 0.57–0.94) compared to adults (OR 0.93, 95% CI 0.87–0.99). However, the meta-regression analyses that controlled the effect of study design, study area, year of data collection, and sample size showed lack of relationship between the age of the study population group with the OR of *Plasmodium* infection reported in each individual study that examined the relationship of the roof or walls of a house, education level, occupation and wealth status with the prevalence or incidence of malaria (S4 Table).

### Publication bias

The funnel plot showing the distributions of the OR of *Plasmodium* infection versus the corresponding standard errors for the ORs among the studies that tested relationship of educational status with malaria prevalence or incidence was symmetrical and the corresponding meta bias tests for the asymmetry was not significant (S2 Fig). However, there was evidence of asymmetry and publication bias among the studies that examined the relationship between housing structure (rooves and walls), income, occupation, and wealth (continuous) with malaria prevalence or incidence (S2 Fig).

### Quality of the studies

The majority of studies employed strong methods for collecting data (n = 67) and for controlling confounders (n = 55). Many studies also demonstrated moderate quality in terms of selecting study participants (n = 60) and blinding of the outcome assessor from knowing the exposure status of participants, and blinding of the study participants to avoid knowing the research question (n = 78). However, 57 studies were cross-sectional in terms of design. Ten studies were rated as being of low quality for lack of controlling confounders, while two studies were rated as low quality on the basis of selecting study participants, and one in terms of the method employed for collecting data. None of the studies were of low quality in terms of blinding of the outcome assessor and the study participants. Overall, quality of the studies based on the criteria’s selection bias, study design, confounders, blinding, data collection method, and withdrawals and drop-outs showed that 20 studies were of strong quality, 54 studies were moderate quality, and 10 studies were rated as low quality (S5 Table).

## Discussion

The current systematic review and meta-analysis suggests that lack of education, low income, poorly constructed houses, and farming are associated with an increased prevalence/incidence of *Plasmodium* infection in SSA. In addition, the review showed a decreased linear trend of *Plasmodium* infection with an increase in the wealth index, which was measured based on household assets ownership, quality of the house, education and occupation. A previous review by Tusting *et al*. (2013) [14] also reported lower odds of *Plasmodium* infection among children who belong to households with low SES group as compared to those who belong to high SES households. However, unlike the current study which examined the linear trend of decrease in *Plasmodium* infection with an increase in the wealth index/socioeconomic group (continuous or categorical) in different population group, the meta-analysis by Tusting *et al*. (2013) [14] limited the comparison to children in the least poor (richest) and poorest socioeconomic groups. In agreement with the current finding, another review by Tusting *et al*. (2015) [15] also showed an increased odds of *Plasmodium* infection among individuals living in houses with mud walls, thatch roof, unscreened window, open eaves and lack of ceilings [15].

The observed association between socioeconomic indicators and *Plasmodium* infection could be due to the effect of SES factors on access and behaviors towards malaria diagnosis, treatment, and prevention measures in SSA. SES indicators may affect behavior and practice of individuals to prevent malaria differently. Education is linked to productivity, capital or potential earning, occupational opportunity, and socialization of individuals. Education also increases knowledge, skills, and ability of the individual to access information that promotes health [74]. Knowledge could lead to a better acceptance and practice of malaria prevention measures. Higher income also provides better housing, schooling, and nutrition that could enhance malaria prevention. Similarly, income level determines the capacity of individuals to buy malaria preventive measures such as insecticide treated nets (ITNs) and indoor residual sprays. Occupation may affect income level, in turn affecting access to malaria preventive measures. Indeed, different studies in SSA have shown an association of mosquito bed net ownership and use with wealth [10,11,112,113], occupation [10,113], and educational status [114]. Another study in Sudan showed an improved use of malaria prevention strategies such as ITNs and house spraying with an increase in household wealth [115].

SES may also affect individuals’ ability to get diagnosed and treated for malaria. Educational status could affect the ability to understand written or verbal information about symptoms, treatment, and transmission mechanisms of malaria. This literacy in turn could affect the practice of individuals to get treated for malaria. Financial resources could also affect individuals’ capability to use available goods and services to treat malaria. For example, the poorest people may not have sufficient money to cover transportation, consultation with healthcare providers, and payment for drugs when they are ill. As a result, people may not seek care. A study in Tanzania found an increased chance of receiving antimalarial drugs in children who had wealthier families compared to poorer families [116].

Some SES indicators may also directly affect the occurrence of malaria. Most malaria transmission in Africa occurs due to mosquitoes resting indoors [117]. The quality of the house thus determines successful entrance of mosquitoes into the house. A house could be constructed without windows or screens, thus exposing individuals to malaria vectors [9]. A study in Gambia found an increased number of indoor residual mosquito vectors in houses made with mud blocks compared to those with concrete ones [118]. The effect of occupation on malaria incidence can also be direct. Some occupations like agriculture may increase contact of individuals with mosquito vectors, increasing the risk of *Plasmodium* infection. In addition, when working in forested areas and migratory activities in the highland fringes, individuals will have less access to healthcare facilities, increasing the risk of *Plasmodium* infection [56].

### Implications for practice

Based on the current review, individuals of low SES have a greater risk of *Plasmodium* infection than those with higher SES. However, current malaria prevention and treatment measures practiced in most endemic regions of SSA have focused on the distribution of ITNs, indoor residual house spray, larval source management, diagnosis, and treatment of cases. SES is often ignored or not sufficiently addressed in malaria control activities. Hence, these measures may not be equally accessible by communities where there is high heterogeneity in SES.

In addition to current malaria control measures, policy makers may wish to consider improving SES of the residents in malaria endemic SSA regions as a supplemental measure [14, 15]. This measure will help to assure availability of the different malaria intervention strategies (including ITN, hose spraying and larval management) to people from low SES households. In effecting this strategy, policy initiatives that target the different components of SES might be important [119,120]. Given the variation in the nature of SES measures that could affect malaria and the complexity in the pathways by which SES underlies malaria, one single policy is unlikely to eliminate health disparities and reduce the malaria burden to the required level in SSA [119,120].

The pro-poor payment strategies, such as vouchers [121], could be one solution in targeting malaria interventions in SSA. Vouchers may help to ensure that malaria treatments be better accessed (purchased) by the communities with low SES. Government or non-government organizations may also support making treatment services accessible financially and geographically to people living in malaria endemic regions. In addition, credit payment methods for treatment costs may be adopted to encourage treatment among the poor [122]. Furthermore, increasing accessibility of public health facilities and drugs without a fee for the poor would supplement malaria control in high prevalence regions [123]. Moreover, creating facilitates to improve community education programs and SES of individuals would effectively combat malaria in SSA. Overall, multisectoral approach policies that support accessibility of malaria diagnosis, treatment and prevention tools to lower SES groups, and maximize individual and organization commitment to strengthen malaria control activities may effectively reduce malaria burden in SSA [124]. Undeniably, in Europe and North America, malaria prevalence reduced as a result of improved SES even before they used specific malaria control strategies [123,125]. Furthermore, the bi-directional nature of the relationship between malaria and SES also suggests controlling malaria could in turn help to improve SES. Thus, evaluation of malaria prevention and treatment policies to combat poverty may be important in SSA.

### Implications for research

The different dimensions of SES-particularly education, income, and housing quality are highly correlated and affect malaria in similar direction. Indeed, the odds of malaria in the current study were greater among individuals with low level of income, and low educational status and among those who were living in poor quality house. Thus, the reported measures of association between educational status and malaria in the original studies might have been confounded or modified by the house nature or income status and vice versa. A composite SES measure, which is based on education, income and house, will help better elucidate the effect of SES on malaria. Future studies may collect data on each component of SES measures-education, income, house quality, assets and occupation, and develop an inclusive valid and reliable measure (SES) to be used for analyzing effect of SES on malaria. In order to test the independent effect of education on malaria risk, intervention studies that involve provision of health education about malaria and evaluation of changes in the risk of infection might be appropriate. In addition, statistical methods used to control for confounding can be applied to examine the independent effect of the different indicators of SES on malaria through observational studies.

### Strengths and limitations

Most effect measures were adjusted and the summary effects were estimated using a random effects model when Moran’s I^2^ values were ≥ 30%. In addition, the log-linear dose-response model was used to estimate the summary effects among studies that determined income status using different cut-off values and those which grouped wealth index in different categories. Moreover, large number of studies were included in the meta-analysis used to estimate the relationship of malaria prevalence or incidence with the nature of the walls and roofs of a house, educational status, and wealth index of individuals. However, this review has some limitations. Due to the cross-sectional nature of some of the studies included in the review and the fact that the nature of relationship of SES and malaria is bidirectional in nature [13], it is not possible to make firm conclusions that SES contributes to the observed high malaria burden in all of the SSA regions. In addition, there was a moderate to high level of heterogeneity in some of the meta-analyses performed. Geographic locations where the studies were conducted, study design, and year of data collection were significant contributors for the increased heterogeneity level among some of the meta-analysis performed. Indeed, sub-group analyses based on geographic region, and study design significantly reduced the heterogeneity level among the studies. In addition, there was a risk of publication bias among the studies included in most of the meta-analyses performed. Moreover, most studies did not consider helminth infection common developing countries that may confound the relationship between SES and malaria [126,127]. Furthermore, pooling of studies that were conducted in urban and rural regions together may not be appropriate. Individuals living in rural areas might be less educated, farmers in their occupation, have low incomes and live in low quality houses made from natural materials thus increasing their risk of getting a malaria infection. Thus, the area where the study was conducted might have confounded the observed relationship between SES and malaria in the current study.

### Conclusions

Lack of education, lower income, poorly constructed houses, and farming may increase the risk of malaria in SSA. Public policy measures that can reduce inequities in health coverage, and improve economic and educational opportunities of the poor may help to reduce the burden of malaria in SSA.

## Supporting information

S1 Table PRISMA checklist. (DOC)

S2 Table Embase literature search strategy. (DOCX)

S3 Table Characteristics of the studies included in this review. (DOCX)

S4 Table Sources of heterogeneity assessment based on multivariable meta-regression analyses. (DOCX)

S5 Table Assessment of the quality of all studies included in the review based on Effective Public Health Practice Project: Quality assessment tool for quantitative studies. (DOCX)

S1 Fig Forest plot showing the relative risk of *Plasmodium* infection among individuals living in house with poor versus good quality, and open versus closed eaves house. (DOCX)

S2 Fig Funnel plots showing the odds ratio of *Plasmodium* infection against the standard errors based on socioeconomic status. (DOCX)
