# Supplementary material for: Improving socioeconomic status may reduce the burden of malaria in sub Saharan Africa: A systematic review and meta-analysis
Source: PLoS One. 2019 Jan 24;14(1):e0211205. doi: 10.1371/journal.pone.0211205 (PMC6345497; doi:10.1371/journal.pone.0211205)
Supplement: S2 Table — (DOCX) [file pone.0211205.s002.docx]

S2 Table. Search details for the Embase

| malaria'/exp OR malaria OR 'plasmodium'/exp OR plasmodium AND ('socioeconomic status'/exp OR 'socioeconomic status' OR 'socioeconomic position'/exp OR 'socioeconomic position' OR 'income'/exp OR income OR 'wealth'/exp OR wealth OR 'poverty'/exp OR poverty OR 'equity'/exp OR equity OR house* OR employment* OR occupation* OR education*) NOT [16-1-2016]/sd |
| --- |
