# Supplementary material for: Improving socioeconomic status may reduce the burden of malaria in sub Saharan Africa: A systematic review and meta-analysis
Source: PLoS One. 2019 Jan 24;14(1):e0211205. doi: 10.1371/journal.pone.0211205 (PMC6345497; doi:10.1371/journal.pone.0211205)
Supplement: S3 Table — (DOCX) [file pone.0211205.s003.docx]

S3 Table. Characteristics of the studies included in this review

| Reference (Study area) | Age group | Sample size | Study design | SES  Indicator | Findings |
| --- | --- | --- | --- | --- | --- |
| Ghebreyesus et al. 2000 [56] (Ethiopia) | Children (<10 years) | 2114 | PC | House | Roof: Thatch vs. corrugate (aRR:1.32, 95% CI: 0.67, 2.63) Roof: Thatch vs earth (aRR:0.47 ,% CI: 0.28, 0.76) Eaves: open vs closed (aRR: 1.85, 95% CI: 1.19, 2.88) Windows: present vs absent (aRR: 1.84, 95% CI: 1.30, 2.63) Animal: yes vs no (aRR: 1.92, 95% CI: 1.29, 2.85) |
| Liu et al. 2013 [70] (Tanzania) | Children (<5 years) | 435 | RCT | House  Education | Good quality vs. poor quality (aRR: 0.32; 95% CI:0.15-0.71) Good quality (iron/tile roofs, concrete/brick walls, closed eaves, screened windows, ceilings) Poor quality (thatched roofs, dirt floors, completely uncovered windows, no ceilings; rough mud walls and open eaves) No schooling vs. formal education (primary or more) (mothers) (RR:1.14, 95% CI: 0.74, 1.76) |
| Wanzirah et al. 2015) [103] (Uganda) | Children | 878 | PC | Wealth  House | Medium vs. low (OR: 0.73, 95% CI:0.62, 0.85)  High vs. low (OR: 0.64, 95% CI:0.54, 0.75)  Floor: earth/sand/dung/stone vs. wood/brick/cement (OR: 3.26, 95% CI: 2.66, 4.00)  Wall: mud vs. cement/wood/metal (OR: 2.91, 95% CI: 2.45, 3.45)  Roof: thatched vs. tiles or metal (OR: 1.73, 95% CI:1.49, 2.02)  Eaves: open vs. closed (OR: 1.83, 95% CI:1.61, 2.09)  Modern (cement/wood/metal wall, tiles or metal roof, closed eaves) vs. traditional (aOR 0.44, 95%CI 0.30, 0.65)  Modern (cement/wood/metal wall, tiles or metal roof, closed eaves) vs. traditional (IRR 0.61, 95%CI: 0.40, 0.91) |
| Nahum et al. 2010 [75] (Benin) | Children (<5 years) | 553 | PC | House | Closed (with a ceiling, possibility of closing windows and doors but lack eaves) vs. open (OR: 0.65, 95% CI: 0.42, 0.99) |
| Osterbauer et al. 2012 [84] (Uganda) | Infants (4 to 6 months) | 600 | CS | House | Good quality (iron sheets roof, bricks or cement walls, and a cement floor) vs. poor quality (aOR:0.25 95% CI: 0.09, 0.72) |
| Nkuo–Akenji et al. 2006 [77] (Cameroon) | All ages | 1454 | CS | House | Wall: wooden plank vs. cement brick (OR: 2.01, 95% CI: 1.44, 2.82) |
| Ouma et al. 2007 [85] (Kenya) | Pregnant women (15 to 42 years) | 685 | CS | House  Occupation | Wall: mud vs. brick/cement (aOR: 1.55, 95% CI: 1.08, 2.23)  Window type: not associated  Maternal occupation (housewife vs. other employment): not associated |
| Coleman et al. 2010 [48] (South Africa) | All ages | 212 | CC | House  Wealth | Wall: mud vs. brick (OR:6.1, 95% CI: 2.26, 16.59)  Roof: Grass vs. corrugated iron (OR: 0.96, 95% CI: 0.44, 2.07)  Window: open vs. closed (OR:4.01, 95% CI 1.32, 12.18).  Poor vs. Poorest (OR: 0.58, 95% CI: 0.23, 1.46)  Less poor vs. Poorest (OR:0.24, 95% CI 0.09, 0.65)  Least poor vs. Poorest (OR:0.27, 95% CI: 0.10, 0.79) |
| De Beaudrap et al.2011 [49] (Uganda) | Children (<5 years) | 2847 | CS | House  Education  Wealth | Good quality (brick walls &iron sheet) vs. poor quality (mud wall & thatched roof) (OR: 0.61, 95% CI: 0.41, 0.91) Education status increases (OR: 0.87, 95% CI: 0.60, 1.26) Wealth index increases (OR: 0.75, 95% CI:0.64, 0.89) |
| Mmbando et al. 2011[23] (Tanzania) | Children and Adolescent (<20 years) | 12298 | CS | House  Wealth | Wall: mud vs bricks (aOR: 1.16, 95% CI:1.01, 1.33) Roof: thatched vs iron (aOR:1.17, 95% CI: 1.04, 1.32) Medium vs. low (aOR:0.99, 95% CI:0.90, 1.08) High vs. low (aOR:0.62, 95% CI:0.52, 0.74) |
| Ayele et al. 2012 [37] (Ethiopia) | All ages | 11601 | CS | House | Wall: wood vs. cement (OR: 0.10, 95% CI: 0.03, 0.25)  Wall: metal vs. cement (OR: 0.54, 95% CI: 0.47, 0.81)  Roof: thatch vs. brick (OR: 3.76, 95% CI:1.24, 9.71)  Roof: mud vs. brick (OR: 0.14, 95% CI:0.05, 0.36)  Floor: earth vs. wood (OR: 5.56, 95% CI: 2.27, 6.67)  Floor: earth vs. cement (OR: 71.42, 95% CI=2.05, 90.9) |
| Yé et al. 2006 [110] (Burkina Faso) | Children (≤5 years) | 661 | CS | House | Roof: mud vs. iron-sheet (OR: 2.6, 95% CI:1.4, 4.7) |
| Temu et al. 2012 [99]  (Mozambique) | Children  (1-15 years) | 8338 | CS | Wealth  Education  House | Poor vs. poorest (aOR: 0.9, 95% CI: 0.7, 1.2) Less poor vs. poorest (aOR: 0.9, 95% CI: 0.7, 1.3) Rich vs. poorest (aOR: 0.5, 95% CI: 0.4, 0.7) None vs. primary/secondary (aOR: 1.67, 95% CI: 1.43, 2.0) None vs. tertiary (aOR: 2.5, 95% CI: 1.67, 3.33) None vs. primary or more (OR: 1.85, 95% CI: 1.67, 2.00) Roof: grass vs. none-grass (aOR: 1.7, 95%CI: 1.3, 2.4) Animals: pigs vs. no pigs (aOR: 3.2, 95%CI: 2.1, 4.9) |
| Oesterholt et al. 2006 [78] (Tanzania) | All ages | 3388 | PC | House | Window: without screen vs. with screens (aOR: 1.54, 95% CI: 1.06, 2.27). |
| Bradley et al. 2013 [41] (Equatorial Guinea) | Children (2-14 years) | 22726 | CS | House | Eaves: Open vs. closed (OR: 1.30, 95% CI:1.13, 1.48)  Window/door: no screen vs. screens (OR: 2.53, 95% CI: 1.69, 3.79) |
| Hagmann et al. 2003 [59] (Principe) | All ages | 1026 | CS | House | Eaves: open vs. closed (RR: 1.57, 95% CI: 1.22, 2.02) |
| Woyessa et al. 2013 [107] (Ethiopia) | All ages | 19207 | CS | Wealth | Middle vs. low (OR: 0.35, 95% CI: 0.25, 0.51)  High vs. low (OR: 0.24, 95% CI: 0.16, 0.36) |
| Ernst et al. 2009 [53] (Kenya) | All ages | 1468 | CC | Education  Wealth  House | None vs. primary/some secondary in progress (OR: 2.01, 95% CI:1.16, 3.05)  None vs. secondary completed (OR: 2.02, 95% CI: 1.08, 3.75)  None vs. primary or secondary (OR: 1.01, 95% CI: 0.75, 1.36)  Poor (2^nd^q) vs. poorest (1^st^ q) (OR: 1.15, 95% CI: 0.85, 1.54)  Less poor (3^rd^q) vs. poorest (1^st^q) (OR: 0.95% CI: 0.69, 1.29)  Rich (4^th^q) vs. poorest (1^st^ q) (OR: 0.85, 95% CI:0.62, 1.16)  Roof: Metal vs not metal roof (OR: 1.43, 95% CI: 1.15, 1.78)  Wall: Mud vs non-mud (OR:0.97, 95% CI: 0.69, 1.35)  Ceiling: absent vs present (OR: 1.53, 95% CI: 1.08, 2.18)  Eaves: open vs closed (OR: 0.89, 95% CI:0.68, 1.17)  Windows: uncovered vs covered (OR:1.06, 95% CI: 0.74, 1.51) |
| Yamamoto et al. 2010 [108]  (Burkina Faso) | Children  (<10 years) | 283 | CC | House  Wealth | Electricity: present vs. absent (OR:2.11, 95% CI:1.02, 4.36)  Wall: Earth bricks vs. cement ( OR:0.22, 95% CI: 0.07, 0.64) Floor: earth vs. stone ( OR: 0.22, 95% CI:0.07, 0.64)  Poor (2^nd^) vs. poorest (1^st^) (OR: 0.83, 95% CI: 0.44, 1.56)  Less poor (3^rd^) vs. poorest (1^st^) (OR: 1.29, 95% CI:0.66, 2.53)  Rich (4^th^) vs. poorest (1^st^) (OR: 2.07, 95% CI: 0.94, 4.58) |
| Peterson et al. 2009 [87] (Ethiopia) | All ages | 1,367 | PC | House | Poor house condition vs. medium or good (IRR:2.15, 95% CI: 0.77, 5.97) Window screened vs unscreened (IRR:2.02, 95% CI: 0.68, 6.02) |
| Ong'echa et al.2006  (Kenya) | Children (0 to 3 years) | 374 | CC | House  Occupation  Education | Wall: mud vs. brick (aOR: 1.96, 95% CI: 0.90, 4.16)  Roof: grass vs. iron (aOR:1.03, 95% CI: 0.50, 2.13)  Window: unscreened vs. screened (aOR: 0.93; 0.46, 1.85)  Window: Screened vs. none (aOR: 0.41, 95% CI: 0.24, 0.70)  Farmer vs. others (mother) (aOR: 2.38, 95% CI: 1.09, 5.26)  Farmer vs. others (head of household) (OR: 0.92, 95% CI: 0.41, 2.04)  Caretaker education (>primary)(aOR: 0.53, 95% CI: 0.26, 1.09) |
| Wolff et al. 2001 (Malawi) | Children (<5 years) | 318 | CS | House | Modern (fired mud bricks, tile roofing, concrete foundation and a pit latrine) vs. traditional (aOR; 0.73, 95% CI: 0.36, 1.40) |
| Kirby et al. 2009(Gambia) | All ages | 500 | RCT | House | Full screened vs unscreened (aOR: 0·94, 95% CI: 0·53, 1·66) |
| Njau et al. 2014 (Angola, Tanzania Uganda) | Children (<5 years) | 10362 | CS | Education  Wealth | No schooling vs. primary (mothers) (OR: 1.42, 95% CI:1.27, 1.59) No schooling vs. beyond primary (mothers) (OR: 2.90, 95% CI:2.37, 3.56) No schooling vs. educated (OR: 1.51, 95% CI:1.36, 1.69)  Poor (2^nd^) vs. poorest (1^st^) (regression coefficient malaria prevalence as a continues; −0.019, p=0.017)) Less poor (3^rd^) vs. poorest (1^st^) (−0.033, p=0.018) Middle (4^th^) vs. poorest (1^st^) (−0.065, p=0.018) Rich (5^th^) vs. poorest (1^st^) (−0.123 (p=0.019) |
| Baragatti et al. 2009 (Burkina Faso) | Children | 3354 | CS | Education | Low education level (no school attendant) vs. high education level (school attended) (OR = 1.33, 95%CI: 1.09, 1.64) |
| Villamor et al. 2003 (Tanzania) | Children (≤5 years) | 546 | CS | Education  House  Occupation | None/illiterate vs. completed elementary (aOR: 1.43, 95% CI:1.00, 2.04) None/illiterate vs. completed secondary (OR: 2.63,95% CI: 1.01, 6.67) None/illiterate vs. elementary or secondary (OR: 2.14, 95% CI: 1.32, 3.46) Animals (pigs): present vs. absent (aOR; 1.66, 95% CI: 1.05, 2.65) Electricity: present vs. absent (OR: 0.63, 95% CI: 0.46, 0.86) Worker vs. house wife (OR: 0.85, 95% CI: 0.60, 1.20) |
| Sezi et al. 2014 (Uganda) | children | 3,847 | CS | Education  Wealth | None/illiterate vs. primary (OR: 1.06, 95% CI: 0.89, 1.26) None/illiterate vs. primary/secondary or more (OR: 3.19, 95% CI: 2.47, 4.12) None/illiterate vs. educated (OR: 1.29, 95% CI: 1.09, 1.52) Poor (2^nd^) vs. poorest (1^st^) (OR: 0.72 , 95% CI: 0.59, 0.87)  Less poor (3^rd^) vs. poorest (1^st^) (OR: 0.60, 95% CI: 0.50, 0.73)  Middle (4^th^) vs. poorest (1^st^) (OR: 0.48, 95% CI: 0.39, 0.58)  Rich (5^th^) vs. poorest (1^st^) (OR: 0.19, 95% CI: 0.15, 0.24) |
| Krefis et al 2010 (Ghana) | Children | 1496 | CS | Education Wealth  House | None/illiterate vs. educated (mothers) (OR: 1.47; 95% CI: 1.18,1.85) Average (2^nd^) vs. poorest (1^st^) (aOR: 0.88, 95% CI: 0.67, 1.15) Rich (3^rd^) vs. poor (1^st^) (aOR: 0.56, 95% CI: 0.42, 0.75)  Wall: Mud/wood vs. Brick/cement ( OR: 0.84, 95% CI: 0.60, 1.16) Electricity: present vs. absent ( OR: 0.72; 95% CI: 0.56, 0.92) |
| Ayi et al. 2010 (Ghana) | All ages | 569 | RCT | Education | Significant decrease (30.9% to 10.3%) in malaria prevalence among children who received education about the disease transmission and prevention, while the disease prevalence increased from 9.5% to 15.9% in those who did not receive the education |
| Deribew et al. 2012 (Ethiopia) | Children (8 to 21 years) | 2410 | RCT | Education | Parents received education about how to use ITN vs. parents who did not have the training (OR = 0.42; 95%CI: 0.32, 0.57) |
| Gahutu et al 2011 (Rwanda) | Children (<5 years) | 749 | CS | Education  Occupation  Income | None (illiterate) vs. primary (father) ( OR: 1.81, 95% CI: 1.18, 2.78)  None (illiterate) vs. secondary or tertiary (OR: 3.45, 95% CI: 1.19, 14.28)  None (illiterate) vs. primary or secondary or tertiary (OR: 1.90, 95% CI: 1.28, 2.83)  Farming/laborer vs. others ( OR: 2.10, 95% CI: 0.99-4.48)  < 5000 RwF vs >= 5000 RwF (AOR: 1.59; 95% CI: 1.05-2.40) |
| Kreuels et al. 2008 (Ghana) | Children (3 months 4 weeks) | 535 | PC | Education Occupation Financial situation | None (illiterate) vs. primary or more (mother) (aRR: 1.30, 95 CI:1.06, 1.59)  Farmer vs non farmer (mother) (aRR: 1.36, 95% CI: 1.18, 1.58) Good (kitchen inside and the house had electricity and piped water) vs poor (lacked one of these parameters) (aRR: 0.66, 95% CI: 0.55, 0.79) |
| Graves et al.2009 (Ethiopia) | All ages | 11538 | CS | Wealth | Poor (2nd) vs. poorest (1st) (OR: 0.86, 95% CI: 0.54, 1.37)  Less poor (3^rd^) vs. poorest (1st) (OR: 0.86, 95% CI: 0.53, 1.40)  Middle (4^th^) vs. poorest (1st) (OR: 1.10, 95% CI: 0.63, 1.92)  Rich (5^th^) vs. poorest (1st) (OR: 0.44, 95% CI: 0.25, 0.77)  Wealth index treated as continuous (aOR:0.79, 95% CI: 0.66, 0.94) |
| Homan et al. 2016 (Kenya) | All ages | 3632 | CS | Wealth  Occupation | Highest vs low (regression coefficient, %malaria prevalence:0.24; P=0.017) [OR: 1.27, 95% CI: 1.08, 1.51]  Outdoor occupation was associated with an increased risk of malaria (regression coefficient, %malaria prevalence =0.57; p<0.0001) |
| Somi et al. 2007 (Tanzania) | All ages | 7,657 | CS | Wealth  House | Poor (2nd) vs. poorest (1st) (OR: 1.11, 95% CI: 0.93, 1.33)  Less poor (3^rd^) vs. poorest (1st) (OR: 0.89, 95% CI: 0.76, 1.05)  Middle (4^th^) vs. poorest (1st) (OR: 0.89, 95% CI: 0.76, 1.05)  Rich (5^th^) vs. poorest (1st) (OR: 0.65, 95% CI: 0.55, 0.77)  Wealth index treated as continuous (OR:0.96, 95% CI: 0.93, 0.99)  Wall: brick/cement vs. mud (malaria prevalence coefficient -0.04, p=0.017) Roof: iron sheet vs. grass/thatched ( malaria prevalence coefficient 0.01, p=0.785) Eves: open vs. closed ( malaria prevalence coefficient 0.01, p=0.562) |
| Clarke et al. 2001(Gambia) | Children (6 months to 5 years) | 1196 | CS | Wealth | Average (2^nd^) vs. low (1^st^) (OR: 0.67, 95% CI: 0.41, 1.09) High (3^rd^) vs. low (1^st^) (OR: 0.42, 95% CI: 0.24, 0.73) |
| Ronald et al. 2006 (Ghana) | Children (1 years 9 years) | 296 | CS | Wealth  Education | Middle (2^nd^) vs. low (1^st^) (OR: 0.40, 95% CI: 0.22, 0.74)  High (3^rd^) vs. low (1^st^) (OR: 0.09, 95% CI: 0.04, 0.21)  Wealth quartiles as continuous (aOR 0.25, 95% CI: 0.14, 0.44)  ≥middle school vs. no (OR: 0.21, 95% CI: 0.12, 0.38) |
| Matthys et al. 2006 (Coˆ te d’Ivoire) | All ages | 672 | CS | Wealth | Poor (2^nd^) vs. poorest (1^st^) (aOR: 0.83, 95% CI: 0.40, 1.55)  Less poor (3^rd^) vs. poorest (1^st^) (aOR: 0.38 , 95% CI: 0.15, 0.78)  Middle (4^th^) vs. poorest (1^st^) (aOR: 1.54, 95% CI: 0.66, 3.08)  Rich (5^th^) vs. poorest (1^st^) (aOR: 0.48, 95% CI: 0.20, 0.97) |
| Winskill et al. 2011 (Tanzania) | Children  (5 to 13 years) | 1438 | CS | House  Wealth | Roof: grass/coconut palm vs. metal sheet (OR: 0.93, 95% CI: 0.69, 1.25)  Wall: mud/wood vs. cement (OR: 1.23, 95% CI: 0.85, 1.77)  Eaves: open vs. closed (OR:1.63, 95% CI:0.73, 3.63)  Wealth quartiles as a continuous ( OR:0.87, 95% CI:0.72, 1.06) |
| Pullan et al. 2010  (Uganda) | All ages | 1,844 | CS | Wealth | Poor (2^nd^) vs. poorest (1^st^) (OR; 0.89, 95% CI: 0.65, 1.21)  Less poor (3^rd^) vs. poorest (1^st^) (OR; 1.01, 95% CI:0.74, 1.38)  Middle (4^th^) vs. poorest (1^st^) (OR; 1.24, 95% CI:0.92, 1.68)  Rich (5^th^) vs. poorest (1^st^) (OR; 0.85, 95% CI:0.70, 1.28) |
| Agomo & Oyibocorresp, 2013 (Nigeria) | Pregnant women | 1084 | CS | Education | Secondary or primary vs. tertiary (OR:1.32, 95% CI:0.77, 2.27) |
| Alemu et al. 2011 (Ethiopia) | Adults | 804 | CS | Education Income | None (illiterate) vs. Primary or more (OR: 1.22, 95 CI: 0.60, 2.48) Monthly income < 31.25USD vs. >62.5USD (aOR: 3.67,95% CI:1.051,12.794) |
| Alemu et al. 2014 (Ethiopia) | Adults | 1455 | CC | Occupation | Farmers vs. others (aOR : 1.40; 95% CI: 1.05, 1.91) |
| Amuta et al. 2014 (Nigeria**)** | Pregnant women | 163 | CS | Education  Occupation | None (illiterate) vs. Primary (OR: 3.71; 95% CI: 0.66, 20.82) None (illiterate) vs. Secondary or tertiary (OR: 3.25; 95% CI: 0.70, 15.07) None (illiterate) vs. Primary or more (OR: 3.32; 95% CI: 0.72, 15.27) Farmers vs. others (aOR =  9.22; 95% CI: 2.00, 42.49) |
| Asante et al. 2013 (Ghana) | Infants | 1855 | PC | Wealth House | Poorest vs. least poor (adjusted HR, 2.21, 95% CI, 1.77, 2.76]. Thatched roof vs. not (adjusted HR, 1.71, 95% CI: 1.51, 1.93]. |
| Asante et al. 2011(Ghana) | Pre-school children | 1671 | CS | Education  Wealth | None (illiterate) vs. Primary (household head) (OR: 1.47, 95% CI: 0.99, 2.13) None (illiterate) vs. Secondary or more (OR: 1.70, 95% CI: 1.32, 2.18) None (illiterate) vs. Primary/secondary or above (OR: 1.65, 95% CI: 1.30, 2.09) Poor (2^nd^) vs. poorest (1^st^) (aOR:0.88, 95% CI:0.60, 1.27) Less poor (3^rd^) vs. poorest (1^st^) (aOR: 0.91, 95% CI: 0.61, 1.34) Middle (4^th^) vs. poorest (1^st^) (aOR:0.59, 95% CI: 0.38, 0.92) Rich (5^th^) vs. poorest (1^st^) (aOR: 0.56, 95% CI: 0.35, 0.91) |
| Bousema et al. 2009 (Tanzani) | All ages | 600 | PC | House  Wealth | Wall: Mud vs. brick (OR:3.12 , 95% CI: 1.49, 6.53)  Roof : thatch vs. iron/tiles (OR:2.01, 95% CI: 1.04, 3.90)  Wealth quintile as continuous (OR: 0.76, 95% CI: 0.58, 1.00) |
| Okebe et al. 2014 (Gambia) | Children | 300 | CC | Education  Occupation  House | No formal vs. primary (mothers) (OR: 1.19, 95% CI:0.55, 2.63) No formal vs. secondary (OR:1.12, 95% CI: 0.62, 2.04) No formal vs. primary or secondary (OR: 1.15, 95% CI: 0.70, 1.89) Farmer (mothers) vs. others (OR: 3.70;95% CI: 1.54, 8.91) Floor: earth vs. tile or cement (OR: 0.56; 95% CI: 0.22, 1.47) Wall: mud vs. cement ( OR: 1.24; 95% CI: 0.76, 2.00) Window: boarded vs. with plane ( OR: 3.95; 95% CI: 2.34, 6.69) |
| Brooker et al 2004 (Kenya) | School age Children | 284 | CC | House  Wealth | Roof: Thatched vs not thatched (OR: 0.98; 95% CI: 0.59, 1.65) Eaves: Open vs closed (OR: 1.10; 95% CI: 0.66, 1.86)  Poor (2^nd^) vs. poorest (1^st^) (OR; 1.30, 95% CI: 0.60, 2.80)  Less poor (3^rd^) vs. poorest (1^st^) (OR; 1.12, 95% CI: 0.52, 2.40)  Middle (4^th^) vs. poorest (1^st^) (OR; 0.53, 95% CI: 0.25, 1.09)  Rich (5^th^) vs. poorest (1^st^) (OR; 0.48, 95% CI: 0.22, 1.06) |
| Bulterys et al 2009 (Zambia) | All ages | 71 | CC | House | Cattle ownership vs. no cattle (aOR: 0.19, 95% CI: 0.05, 0.69) |
| Chaponda et al 2015 (Zambia) | Pregnant women | 1086 | CS | Wealth | Poor (2^nd^) vs. poorest (1^st^) (aOR: 0.73, 95% CI: 0.50, 1.09)  Less poor (3^rd^) vs. poorest (1^st^) (aOR: 0.76, 95% CI: 0.51, 1.15)  Middle (4^th^) vs. poorest (1^st^) (aOR: 0.90, 95% CI: 0.59, 1.38)  Rich (5^th^) vs. poorest (1^st^) (aOR: 0.52, 95% CI: 0.35, 0.80) |
| Charlwood et al. 2015 (Mozambique) | Children & adult | 500 | PC | House | Roof: Thatch/grass/leaf vs. corrugated iron/cement (aOR:2.16, 95%CI:1.41, 3.38)  Wall: Cane/palm/trunks/mud/dirt vs. bricks/tiles/cement (aOR: 0.52, 95 CI: 0.29, 0.90)  Animal present vs. no animal (OR:1.02; 95% CI: 0.84, 1.24) |
| Clerk et al. 2009 (Ghana) | Pregnant women | 3642 | RCT | Education | Some level of education associated with a reduction in the risk of high-density parasitaemia (aOR 0.72, 95% CI 0.54–0.96) (reference group not indicated |
| De Castro & Fisher, 2012 (Tanzania) | Pres-school (<5 years) | 5,584 | CS | Education  Occupation  Wealth | Secondary level vs higher (parents) (OR:0.002; 95% CI:-0.047, 0.043)  Farming vs non-farming (parents) (OR:0.009; 95% CI:-0.014, 0.032)  Household’s wealth status was not associated with odds of malaria (regression coefficient :−0.007; 95% CI:-0.04, 0.027) [OR:0.99; 95% CI:0.96, 1.03) (wealth index treated as continuous) |
| Elmardi et al. 2011 (Sudan) | All ages | 26,471 | CS | Wealth | Poor (2^nd^) vs. poorest (1^st^) (OR: 1.23, 95% CI: 0.97, 1.56)  Less poor (3^rd^) vs. poorest (1^st^) (OR: 0.70, 95% CI: 0.54, 0.91)  Middle (4^th^) vs. poorest (1^st^) (OR: 0.40, 95% CI: 0.30, 0.55)  Rich (5^th^) vs. poorest (1^st^) (OR: 0.15, 95% CI: 0.09, 0.23) |
| Florey et al. 2012 (Kenya) | Children | 561 | CS | Wealth | Wealth index treated as continuous: (aOR:0.70, 95% CI: 0.55, 0.90) |
| Gosoniu etal. 2012 (Tanzania) | Pre-school | 6360 | CS | Wealth | Poor (2^nd^) vs. poorest (1^st^) (OR: 0.89, 95% CI: 0.71, 1.10)  Less poor (3^rd^) vs. poorest (1^st^) (OR: 0.81, 95% CI: 0.65, 1.02)  Middle (4^th^) vs. poorest (1^st^) (OR: 0.55, 95% CI: 0.42, 0.72)  Rich (5^th^) vs. poorest (1^st^) (OR: 0.28, 95% CI: 0.17, 0.48) |
| Haji et al. 2016 (Ethiopia) | Children | 830 | CS | Wealth | Medium vs low (aOR:1.51, 95% CI:0.51, 4.45)  High vs low (aOR:0.93, 95% CI:0.35, 2.45) (not indicated how index estimated) |
| Houngbedji et al. 2015 (Côte d’Ivoire) | Children | 5,122 | CS | Wealth | Poor (2^nd^) vs. poorest (1^st^) (aOR: 0.90, 95% CI: 0.73, 1.11)  Less poor (3^rd^) vs. poorest (1^st^) (aOR: 0.95, 95% CI: 0.77, 1.18)  Middle (4^th^) vs. poorest (1^st^) (aOR: 0.81, 95% CI: 0.66, 1.01)  Rich (5^th^) vs. poorest (1^st^) (aOR; 0.89,95% CI: 0.72, 1.09) |
| Kalu et al. 2012 (Nigeria) | All ages | 632 | CS | Occupation | Farmers vs non-farmers (civil servants, entrepreneurs, traders, students) (OR: 1.35, 95% CI: 0.72, 2.52) |
| Keating et al. 2009 (Zambia) | Pre-school age | 618 | CS | Wealth | Poor (2^nd^) vs. poorest (1^st^) (OR: 1.69, 95% CI: 0.62, 4.57)  Less poor (3^rd^) vs. poorest (1^st^) (OR: 0.78, 95% CI: 0.32, 1.88)  Middle (4^th^) vs. poorest (1^st^) (OR: 0.68, 95% CI: 0.26, 1.78)  Rich (5^th^) vs. poorest (1^st^) (OR: 0.36, 95% CI: 0.12, 1.09) |
| Kibret et al. 2009 (Ethiopia) | All ages | 2435 | PC | House Education Occupation Income | Corrugated iron vs grass (%malaria prevalence coefficient: -0.57, p<0.01) Education (%malaria prevalence coefficient : -0.369, p>0.05) Occupation (%malaria prevalence coefficient: -0.37, p>0.05) Income (%malaria prevalence coefficient: 0.0002, p<0.01) |
| Knoblauch et al. 2014 (Côte d’Ivoire) | Preschool age & mothers | 574 | CS | Education (mothers) | No formal vs. educated (mothers) (aOR: 1.15, 95% CI: 0.64, 2.08) |
| Koram et al. 1995 (Gambia) | Children | 350 | CC | House  Education | Wall: mud vs. cement/brick (OR: 1.73, 95% CI: 1.09, 2.81)  Ceiling: absent vs present (OR: 1.41, 95% CI: 1.01, 1.97)  No association between the risk of malaria and education level of parents or guardians |
| Kyu et al.2013 (Nigeria) | Women (15-49years) & children (<5 years) | 4082 | CS | Wealth Education | Wealth continuous (aOR: 0.81, 95% CI: 0.66, 1.00) Education (years of schooling) (aOR: 0.98; 95% CI: 0.95, 1.01) |
| Mathanga et al. 2015 (Malawi) | Children  (5-18 years) | 2626 | CS | Education  Wealth | No formal vs. some/completed primary ( aOR:1.12, 95% CI: 0.88, 1.43)  No formal vs. some/completed secondary (aOR: 1.54, 95%CI: 1.09, 2.7)  No formal vs. educated (OR: 1.12, 95% CI: 0.9, 1.38)  Poor (2^nd^) vs. poorest (1^st^) (OR:1.08, 95% CI:0.82, 1.42)  Less poor (3^rd^) vs. poorest (1^st^) (OR: 1.30, 95% CI:0.98, 1.72)  Middle (4^th^) vs. poorest (1^st^) (OR:1.26, 95% CI:0.94, 1.70)  Rich (5^th^) vs. poorest (1^st^) (OR: 0.74, 95% CI:0.55, 1.00) |
| Mbu et al. 2014 (Camerroon) | Pregnant women (15-39 years) | 332 | Nested CC | Education | Primary vs. secondary/tertiary (OR: 3.69, 95% CI: 1.58, 4.59)  Secondary vs. tertiary (OR: 1.52, 95% CI: 0.88, 2.620 |
| Mmbando et al 2009 (Tanzania) | Children & adolescents | 6375 | CS | Wealth | Low vs. high (aOR: 2.02, 95%CI: 1.69, 2.41)  Medium vs. high ( aOR: 2.47, 95%CI: 2.05, 2.99) |
| Njau et al 2006 (Tanzania) | All ages | 3693 | CS | Wealth | Middle vs. low (OR: 1.05, 95% CI:0.87, 1.28)  High vs. low (OR: 0.65, 95% CI:0.53,0.8) |
| Omer et al. 2011 (Sudan) | Pregnant women | 836 | CS | Income | Low vs high (aOR: 2.3, 95% CI: 1.3, 4.0) |
| Omokanye et al. 2012 (Nigeria) | Pregnant women  (28.9±4.7) | 412 | CS | Education | No formal vs. primary (OR:1.56, 95% CI: 0.17, 14.27)  No formal vs. secondary/tertiary (OR:29.09, 95% CI: 3.58, 236.17)  No formal vs. primary or more (OR: 30.2, 95% CI:3.72, 244.89) |
| Onyido et al. 2011 (Nigeria) | All ages | 178 | CS | Education  Occupation | Malaria prevalence was lower among individuals with tertiary education level compared to those with no formal, primary or secondary education level No significant difference in the prevalence of malaria between farmers and other occupations |
| Peterson et al. 2009 [86] (Ethiopia) | All ages | 8,088 | PC | House | Poor house vs good house (aRR: 2.0, 95% CI:1.4, 2.9)  House (not defined) |
| Rulisa et al. 2013 [90] (Rwanda) | Children | 769 | CS | House | Wall: wood/mud/tent vs. bricks/stones (aOR: 1.29, 95% CI: 1.08, 1. 53)  Roof: grass thatched/tent/others vs. corrugated iron sheet (aOR: 0.84, 95% CI: 0.64, 1.10) |
| Sintasath et al 2005 [92] (Eretria) | All ages | 12,937 | CS | House | Wall: mud vs. other (aOR: 1.60, 95% CI: 1.15, 2.24)  Eaves: open vs. closed ( aOR: 1.17, 95% CI: 0.84, 1.63) |
| Skarbinski et al. 2012 [93] (Malawi) | Preschool children | 899 | CS | Wealth  House | Poor (2^nd^) vs. poorest (1^st^) (OR: 0.92, 95% CI 0.63, 1.34) Less poor (3^rd^) vs. poorest (1^st^) (OR: 0.96, 95% CI: 0.64, 1.45) Middle (4^th^) vs. poorest (1^st^) (OR: 0.50, 95% CI: 0.31, 0.81) Rich (5^th^) vs. poorest (1^st^) (OR: 0.30, 95% CI: 0.17, 0.52) Wall: Mud vs. brick/stone (OR: 1.17, 95% CI: 0.95, 1.44) Roof: grass/thatched vs. iron sheet (OR: 1.04, 95% CI: 0.73, 1.46) |
| Snyman et al. 2015 [94] (Uganda) | Pre-school Children | 515 | PC | House  Wealth  Education | Modern (non-earth floors, non-thatched roofs, non-mud walls) vs. traditional (aIRR:0.54, 95% CI: 0.39, 0.78) Window: present vs. absent (aIRR:0.71, 95% CI: 0.61, 0.84) Eaves: present vs. absent (aIRR:0.81, 95% CI: 0.69, 0.96) Middle vs. lowest (aIRR:0.91, 95% CI: 0.76, 1.1) Highest vs. lowest (aIRR: 0.86, 95% CI: 0.72, 1.03) More than primary vs non or primary (aIRR: 0.78, 95% CI: 0.62, 0.99) |
| Somi et al. 2008 [96] (Tanzania) | All ages | 1577 | CS | Wealth index (SES)  House | Coefficient (quantiles as continues & malaria prevalence continuous) −0.003, p<0.05)  Roof: manufactured (tiles, iron) vs. others (coefficient, malaria prevalence -0.140, p<0.05)  Wall: manufactured (cement/brick) vs others. (coefficient, malaria prevalence-0.1, p<0.05) Eaves: present vs. absent (coefficient, malaria prevalence-0.04, p<0.05) |
| Sonko et al 2014 [97] (Gambia) | All ages | 5541 | CS | Wealth  House | Poor (2^nd^) vs. poorest (1^st^) (aOR:0.4, 95% CI: 0.2, 0.7)  Less poor (3^rd^) vs. poorest (1^st^) (aOR: 0.5, 95% CI: 0.3, 0.9)  Middle (4^th^) vs. poorest (1^st^) (aOR: 0.3, 95% CI: 0.1, 0.6)  Rich (5^th^) vs. poorest (1^st^) (aOR: 0.1, 95% CI: 0.04, 0.3)  Wall: Cane/palm/trunks, mud/dirt) vs. Bricks/cement blocks (aOR: 1.8, 95% CI: 1.1, 3.1)  Floor: Earth/sand/dung/palm bamboo) vs cement/tile) (aOR: 2.0, 95% CI: 1.4, 3.0)  Roof: Thatch/leaf vs. Sod/metal corrugated iron/cement concrete (aOR: 2.2, 95% CI: 1.4, 3.3)  Window; poor (No window at all or window without glasses, screen, curtain, or shutters) vs. good (window with at least glasses, screen, curtain, or shutters) (aOR: 1.8 95% CI: 1.1, 3.0) |
| Steinhardt et al. 2013 [98] (Uganda) | Pre-school children | 1770 | CS | Wealth index | Poor (2^nd^) vs. poorest (1^st^) (aOR: 0.94, 95% CI: 0.80, 1.11)  Less poor (3^rd^) vs. poorest (1^st^) (aOR: 0.77, 95% CI: 0.63, 0.94)  Middle (4^th^) vs. poorest (1^st^) (aOR: 0.73, 95% CI: 0.61, 0.88)  Rich (5^th^) vs. poorest (1^st^) (aOR: 0.59, 95% CI: 0.46, 0.77) |
| Tonga et al 2013 [100] (Cameroon) | Pregnant | 201 | CS | Education Income | No education/primary vs. secondary or more (OR: 0.8 (0.4–1.6) Monthly income: <28000 FCFA vs. >28000 FCFA (OR: 3.9 (1.3–11.5) |
| Townes et al. 2013 [101] (Malawi) | Pre-school children | 390 | CS | House | Wall: mud vs. cement/brick (OR: 0.67, 95% CI: 0.44, 1.03)  Roof: thatch vs. metal (OR: 1.99, 95% CI: 1.12, 3.51)  Window: glass vs. other (OR: 0.84, 95% CI: 0.52, 1.35) |
| West et al. 2013 [104] (Tanzania) | Children | S1:5142  S2:4306 | CS | Wealth | Poor (2^nd^) vs. poorest (1^st^) (OR: 0.79, 95%CI: 0.64, 0.98)  Less poor (3^rd^) vs. poorest (1^st^) (OR: 0.62, 95%CI: 0.50, 0.77)  Middle (4^th^) vs. poorest (1^st^) (OR: 0.42, 95%CI: 0.34, 0.53)  Rich (5^th^) vs. poorest (1^st^) (OR: 0.31, 95%CI: 0.24, 0.39)  Wealth quartiles as a continues (aOR: 0.91, 95% CI: 0.83, 0.99) |
| Yatich et al. 2009 [109] (Ghana) | Pregnant women | 746 | CS | Education Income | No formal vs. primary/secondary/tertiary (aOR; 0.8, 95% CI: 0.5, 1.2) < 200,000 cedis vs. >200,000 cedis (aOR; 1.8, 95% CI:1.2 – 2.9) |

aOR: adjusted odds ratio; aRR: adjusted relative risk; CC: Case Control; CS: Cross-sectional; NA: not available/not provided/not mentioned/not speciﬁed; OR: odds ratio; RCT: randomized control trial; RR: Relative risk; PC: Prospective control
