# Supplementary material for: Improving socioeconomic status may reduce the burden of malaria in sub Saharan Africa: A systematic review and meta-analysis
Source: PLoS One. 2019 Jan 24;14(1):e0211205. doi: 10.1371/journal.pone.0211205 (PMC6345497; doi:10.1371/journal.pone.0211205)
Supplement: S4 Table — (DOCX) [file pone.0211205.s004.docx]

S4 Table. Sources of heterogeneity assessment based on multivariable meta-regression analyses

| SES characteristics | Comparison | Factors | Meta-regression coefficient | *p*-value | Bonferroni corrected *p* value |
| --- | --- | --- | --- | --- | --- |
| House (wall) | Mud vs brick/cement/metal | Study region | 0.31 | 0.152 | 0.912 |
|  |  | Age | -0.01 | 0.902 | 1.000 |
|  |  | Study design | 0.33 | 0.134 | 0.804 |
|  |  | Study quality | 0.35 | 0.245 | 1.000 |
|  |  | Year of data collection | -0.02 | 0.528 | 1.000 |
|  |  | Sample size | <0.001 | 0.784 | 1.000 |
| House (roof) | Thatch/grass\| vs iron sheet\| corrugate \|metal | Study region | **-0.39** | **0.045** | 0.270 |
|  |  | Age | 0.12 | 0.246 | 1.000 |
|  |  | Study design | 0.34 | 0.116 | 0.696 |
|  |  | Study quality | 0.23 | 0.445 | 1.000 |
|  |  | Year of data collection | 0.002 | 0.956 | 1.000 |
|  |  | Sample size | 0.00003 | 0.237 | 1.000 |
| Education | Uneducated vs educated | Study region | **0.21** | **0.002** | **0.012** |
|  |  | Age | **-0.15** | **0.034** | 0.204 |
|  |  | Study design | -0.25 | 0.128 | 0.768 |
|  |  | Study quality | -0.03 | 0.772 | 1.000 |
|  |  | Year of data collection | -0.02 | 0.165 | 0.990 |
|  |  | Sample size | <0.001 | 0.108 | 0.648 |
| Education | Primary vs secondary or more | Study region | **0.63** | **0.040** | 0.240 |
|  |  | Age | -0.26 | 0.139 | 0.834 |
|  |  | Study design | **0.79** | **0.042** | 0.252 |
|  |  | Study quality | 0.49 | 0.107 | 0.642 |
|  |  | Year of data collection | 0.02 | 0.413 | 1.000 |
|  |  | Sample size | **0.00001** | **0.016** | 0.096 |
| Occupation | Farmers vs non-farmers | Study region | -0.38 | 0.150 | 0.750 |
|  |  | Age | 0.08 | 0.434 | 1.000 |
|  |  | Study design | -0.06 | 0.921 | 1.000 |
|  |  | Year of data collection | 0.13 | 0.427 | 1.000 |
|  |  | Sample size | -0.001 | 0.450 | 1.000 |
| Income | Low vs high | Study region | -0.19 | 0.623 | 1.000 |
|  |  | Age | 0.03 | 0.923 | 1.000 |
|  |  | Study design | -0.06 | 0.921 | 1.000 |
|  |  | Year of data collection | 0.01 | 0.942 | 1.000 |
|  |  | Sample size | -0.001 | 0.118 | 0.590 |
| Wealth | Continuous | Study region | **0.49** | **0.039** | 0.234 |
|  |  | Age | 0.19 | 0.061 | 0.366 |
|  |  | Study design | -0.15 | 0.281 | 1.000 |
|  |  | Year of data collection | **0.10** | **0.041** | 0.246 |
|  |  | Quality | -0.29 | 0.278 | 1.000 |
|  |  | Sample size | <0.001 | 0.662 | 1.000 |

Study region (Africa vs Asia), Methods for diagnosis of *Plasmodium* infection (PCR vs microscope), Study design (Case control vs cross-sectional), Age (All ages vs Adult vs Children)
