# Supplementary material for: Improving socioeconomic status may reduce the burden of malaria in sub Saharan Africa: A systematic review and meta-analysis
Source: PLoS One. 2019 Jan 24;14(1):e0211205. doi: 10.1371/journal.pone.0211205 (PMC6345497; doi:10.1371/journal.pone.0211205)
Supplement: S5 Table — (DOCX) [file pone.0211205.s005.docx]

S6 Table. Assessment of the quality of all studies included in the review based on Effective Public Health Practice Project: Quality assessment tool for quantitative studies

| Study  no. | Author (Year) | Selection  bias | Study  design | Confounders | Blinding | Data collection methods | Withdrawals and drop-outs | Final  rating |
| --- | --- | --- | --- | --- | --- | --- | --- | --- |
| 1 | Ghebreyesus et al. 2000 | 1 | 2 | 1 | 2 | 2 | 1 | 1 |
| 2 | Liu et al. 2013 | 1 | 1 | 1 | 1 | 1 | 1 | 1 |
| 3 | Wanzirah et al. 2015 | 1 | 2 | 1 | 2 | 1 | 1 | 1 |
| 4 | Nahum et al. 2010 | 2 | 2 | 1 | 2 | 1 | 2 | 1 |
| 5 | Osterbauer et al. 2012 | 2 | 3 | 1 | 2 | 1 | NA | 2 |
| 6 | Nkuo–Akenji et al. 2006 | 2 | 3 | 3 | 2 | 1 | NA | 3 |
| 7 | Ouma et al. 2007 | 1 | 3 | 1 | 2 | 1 | NA | 2 |
| 8 | Coleman et al. 2010 | 2 | 2 | 1 | 1 | 3 | 2 | 2 |
| 9 | De Beaudrap et al. 2011 | 1 | 3 | 1 | 2 | 2 | NA | 2 |
| 10 | Mmbando et al. 2011 | 2 | 3 | 1 | 2 | 1 | NA | 2 |
| 11 | Ayele et al. 2013 | 2 | 3 | 1 | 2 | 1 | NA | 2 |
| 12 | Yé et al. 2006 | 2 | 3 | 1 | 2 | 1 | NA | 2 |
| 13 | Temu et al. 2012 | 2 | 3 | 1 | 2 | 1 | NA | 2 |
| 14 | Oesterholt et al. 2006 | 2 | 2 | 1 | 2 | 1 | 3 | 2 |
| 15 | Bradley et al. 2013 | 2 | 3 | 1 | 2 | 2 | NA | 2 |
| 16 | Hagmann et al. 2003 | 2 | 3 | 3 | 2 | 2 | NA | 3 |
| 17 | Woyessa et al. 2013 | 2 | 3 | 1 | 1 | 1 | NA | 2 |
| 18 | Ernst et al. 2009 | 2 | 2 | 1 | 2 | 2 | 2 | 1 |
| 19 | Yamamoto et al. 2010 | 2 | 2 | 1 | 1 | 2 | 2 | 1 |
| 20 | Peterson et al. 2009 | 2 | 2 | 1 | 2 | 2 | 3 | 2 |
| 21 | Wolff et al. 2001 | 2 | 3 | 1 | 2 | 1 | NA | 2 |
| 22 | Kirby et al. 2009 | 1 | 1 | 1 | 2 | 1 | 1 | 1 |
| 23 | Njau et al. 2014 | 2 | 3 | 1 | 2 | 2 | NA | 2 |
| 24 | Baragatti et al. 2009 | 2 | 3 | 1 | 1 | 1 | NA | 2 |
| 25 | Villamor et al. 2003 | 2 | 3 | 2 | 2 | 2 | NA | 2 |
| 26 | Sezi, 2014 | 2 | 3 | 3 | 2 | 1 | NA | 3 |
| 27 | Krefis et al. 2010 | 2 | 3 | 1 | 2 | 1 | NA | 2 |
| 28 | Ayi et al. 2010 | 1 | 1 | 1 | 2 | 1 | 2 | 1 |
| 29 | Deribew et al. 2012 | 2 | 1 | 1 | 2 | 2 | 1 | 1 |
| 30 | Gahutu et al. 2011 | 2 | 3 | 1 | 2 | 1 | NA | 2 |
| 31 | Kreuels et al. 2008 | 2 | 1 | 1 | 1 | 1 | 1 | 1 |
| 32 | Graves et al. 2009 | 1 | 3 | 1 | 2 | 1 | NA | 2 |
| 33 | Homan et al. 2016 | 2 | 3 | 1 | 2 | 2 | NA | 2 |
| 34 | Somi et al. 2007 | 2 | 3 | 1 | 2 | 1 | NA | 2 |
| 35 | Clarke et al. 2001 | 3 | 3 | 3 | 2 | 1 | NA | 3 |
| 36 | Ronald et al. 2006 | 2 | 3 | 1 | 2 | 1 | NA | 2 |
| 37 | Winskill et al 2011 | 2 | 3 | 1 | 2 | 1 | NA | 2 |
| 38 | Matthys et al. 2011 | 1 | 3 | 1 | 2 | 1 | NA | 2 |
| 39 | Pullan et al 2010 | 1 | 3 | 2 | 2 | 1 | NA | 2 |
| 40 | Agomo &Oyibocorresp, 2013 | 2 | 3 | 2 | 2 | 1 | NA | 2 |
| 41 | Alemu et al. 2011 | 2 | 3 | 1 | 2 | 1 | NA | 2 |
| 42 | Alemu et al. 2014 | 2 | 2 | 1 | 2 | 1 | 1 | 1 |
| 43 | Amuta et al. 2014 | 2 | 3 | 3 | 2 | 1 | NA | 3 |
| 44 | Asante et al. 2013 | 1 | 2 | 1 | 2 | 1 | 1 | 1 |
| 45 | Asante et al. 2011 | 1 | 3 | 1 | 2 | 1 | NA | 2 |
| 46 | Bousema et al. 2009 | 2 | 2 | 2 | 2 | 2 | 3 | 2 |
| 47 | Brooker et al 2004 | 2 | 2 | 1 | 2 | 1 | NA | 1 |
| 48 | Bulterys et al 2009 | 1 | 2 | 2 | 2 | 1 | NA | 1 |
| 49 | Chaponda et al 2015 | 2 | 3 | 2 | 2 | 1 | NA | 2 |
| 50 | Charlwood et al. 2015 | 2 | 1 | 1 | 2 | 1 | 2 | 1 |
| 51 | Clerk et al. 2009 | 2 | 1 | 1 | 2 | 1 | 2 | 1 |
| 52 | De Castro & Fisher, 2012 | 1 | 3 | 2 | 2 | 2 | NA | 2 |
| 53 | Elmardi et al. 2011 | 1 | 3 | 2 | 2 | 2 | NA | 2 |
| 54 | Florey et al. 2012 | 3 | 3 | 1 | 2 | 1 | NA | 3 |
| 55 | Gosoniu, etal. 2012 | 2 | 3 | 1 | 2 | 1 | NA | 2 |
| 56 | Haji et al. 2016 | 2 | 3 | 1 | 2 | 1 | NA | 2 |
| 57 | Houngbedji et al. 2015 | 1 | 3 | 1 | 2 | 1 | NA | 2 |
| 58 | Kalu et al. 2012 | 2 | 3 | 3 | 2 | 1 | NA | 3 |
| 59 | Keating et al. 2009 | 1 | 3 | 2 | 2 | 1 | NA | 2 |
| 60 | Kibret et al. 2009 | 2 | 2 | 3 | 2 | 1 | 2 | 2 |
| 61 | Knoblauch et al. 2014 | 2 | 3 | 1 | 2 | 2 | NA | 2 |
| 62 | Koram et al. 1995 | 2 | 2 | 2 | 2 | 1 | 2 | 2 |
| 63 | Kyu et al.2013 | 1 | 3 | 2 | 2 | 1 | NA | 2 |
| 64 | Mathanga et al. 2015 | 1 | 3 | 1 | 2 | 1 | NA | 2 |
| 65 | Mbu et al. 2014 | 2 | 2 | 2 | 2 | 1 | NA | 1 |
| 66 | Mmbando et al 2009 | 2 | 3 | 2 | 2 | 1 | NA | 2 |
| 67 | Njau et al 2006 | 1 | 3 | 3 | 2 | 1 | NA | 3 |
| 68 | Okebe et al. 2014 | 2 | 2 | 1 | 2 | 1 | NA | 1 |
| 69 | Omer et al. 2011 | 2 | 3 | 2 | 2 | 1 | NA | 2 |
| 70 | Omokanye et al. 2012 | 2 | 3 | 2 | 2 | 1 | NA | 2 |
| 71 | Ong’echa et al. 2006 | 2 | 3 | 1 | 2 | 1 | NA | 2 |
| 72 | Onyido et al. 2011 | 2 | 3 | 3 | 2 | 1 | NA | 3 |
| 73 | Peterson et al. 2009 | 2 | 2 | 2 | 2 | 1 | NA | 1 |
| 74 | Rulisa et al. 2013 | 2 | 3 | 1 | 2 | 1 | NA | 2 |
| 75 | Sintasath et al 2005 | 2 | 3 | 1 | 2 | 2 | NA | 2 |
| 76 | Skarbinski et al. 2012 | 2 | 3 | 2 | 2 | 1 | NA | 2 |
| 77 | Snyman et al. 2015 | 2 | 1 | 1 | 2 | 1 | 1 | 1 |
| 78 | Somi et al. 2008 | 2 | 3 | 1 | 2 | 1 | NA | 2 |
| 79 | Sonko et al. 2014 | 2 | 3 | 1 | 2 | 1 | NA | 2 |
| 80 | Steinhardt et al. 2013 | 1 | 3 | 1 | 2 | 1 | NA | 2 |
| 81 | Tonga et al 2013 | 2 | 3 | 2 | 2 | 1 | NA | 2 |
| 82 | Townes et al. 2013 | 2 | 3 | 3 | 2 | 1 | NA | 3 |
| 83 | West et al. 2013 | 1 | 3 | 2 | 2 | 1 | NA | 2 |
| 84 | Yatich et al. 2009 | 2 | 3 | 1 | 2 | 1 | NA | 2 |
