# Supplementary material for: Improving socioeconomic status may reduce the burden of malaria in sub Saharan Africa: A systematic review and meta-analysis
Source: PLoS One. 2019 Jan 24;14(1):e0211205. doi: 10.1371/journal.pone.0211205 (PMC6345497; doi:10.1371/journal.pone.0211205)
Supplement: S1 Fig — (DOCX) [file pone.0211205.s006.docx]

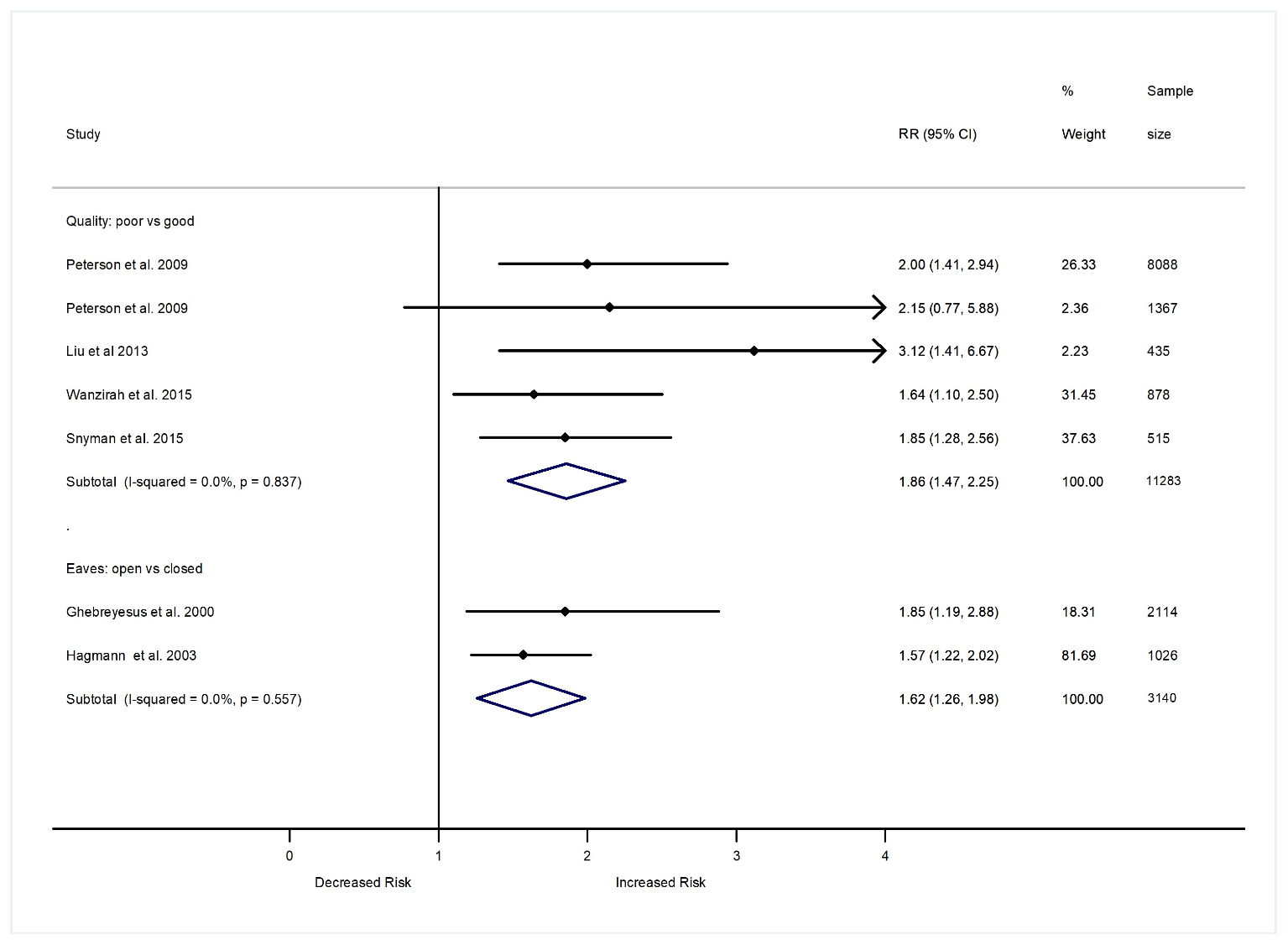


S1 Fig. Forest plot showing the relative risk of *Plasmodium* infection among individuals living in house with poor versus good quality, and open versus closed eaves house
